# Temporal analysis shows relaxed genetic erosion following improved stocking practices in a subarctic transnational brown trout population

**DOI:** 10.1038/s41598-021-96681-1

**Published:** 2021-08-30

**Authors:** Cornelya F. C. Klütsch, Simo N. Maduna, Natalia Polikarpova, Kristin Forfang, Benedicte Beddari, Karl Øystein Gjelland, Paul Eric Aspholm, Per-Arne Amundsen, Snorre B. Hagen

**Affiliations:** 1 Division of Environment and Natural Resources, Norwegian Institute of Bioeconomy Research (NIBIO), Svanhovd, Norway; 2 Pasvik Strict Nature Reserve, 184424 Rajakoski, Murmansk Region Russia; 3 Norwegian Institute for Nature Research (NINA), 9296 Tromsø, Norway; 4 Division of Forest and Forest Resources, Norwegian Institute of Bioeconomy Research (NIBIO), Svanhovd, Norway; 5 Department of Arctic and Marine Biology, Faculty of Biosciences, Fisheries and Economics, UiT The Arctic University of Norway, Tromsø, Norway

**Keywords:** Ecological genetics, Molecular ecology, Biological techniques, Genetics, Zoology, Ecology

## Abstract

Maintaining standing genetic variation is a challenge in human-dominated landscapes. We used genetic (i.e., 16 short tandem repeats) and morphological (i.e., length and weight) measurements of 593 contemporary and historical brown trout (*Salmo trutta*) samples to study fine-scale and short-term impacts of different management practices. These had changed from traditional breeding practices, using the same broodstock for several years, to modern breeding practices, including annual broodstock replacement, in the transnational subarctic Pasvik River. Using population genetic structure analyses (i.e., Bayesian assignment tests, DAPCs, and PCAs), four historical genetic clusters (E2001A-D), likely representing family lineages resulting from different crosses, were found in zone E. These groups were characterized by consistently lower genetic diversity, higher within-group relatedness, lower effective population size, and significantly smaller body size than contemporary stocked (E2001E) and wild fish (E2001F). However, even current breeding practices are insufficient to prevent genetic diversity loss and morphological changes as demonstrated by on average smaller body sizes and recent genetic bottleneck signatures in the modern breeding stock compared to wild fish. Conservation management must evaluate breeding protocols for stocking programs and assess if these can preserve remaining natural genetic diversity and morphology in brown trout for long-term preservation of freshwater fauna.

## Introduction

Standing genetic variation is a key factor for the preservation of ecological and evolutionary functions in natural populations, as it is linked to population fitness and adaptive potential^1,2^. Hence, standing genetic variation is the basis for responses to anthropogenic pressures and environmental change, and it ensures the long-term survival of species^1–3^. Spatial genetic analyses have greatly improved our understanding of intraspecific genetic variation, including phenomena like gene flow and connectivity and/or isolation of populations^1,3,4^. In comparison, temporal changes in genetic variation are less studied, despite their importance for predicting impacts of anthropogenic activities and environmental disturbances on natural populations^1,3,5,6^. Recent conservative estimates put the global loss of intraspecific genetic variation at 6% since the industrial revolution^2^. This coincides with estimates that the rate of local population extinction is at least three orders of magnitude higher than species extinctions^7^.

Temporal fluctuations in population size and genetic diversity can cause shifts in allele frequencies that are not completely captured by spatial analyses. Temporal study designs can enhance our understanding of demographic fluctuations, like changes in effective population size and spatial genetic differentiation^3,6^. In fish species, the analysis of temporal genetic changes has attracted interest for studying genetic depletion/replacement and loss of locally adapted populations^5,8^. Further, changes in population genetic structure caused by release of translocated broodstocks, stocking, and accidental escapes from fish farms has been in the focus of fishery research^5,8^. Other anthropogenic impacts, like overharvesting and the destruction of natural spawning and nursery areas, can lower reproduction resulting in decreasing population sizes and thus, decreased genetic diversity over time^4^. In addition, hydroelectric dams increase isolation and genetic divergence between river zones^4^. Negative impacts of anthropogenic infrastructure may have been unforeseen in some cases, but often, such as with construction of hydroelectric dams, they were to some extent foreseen. Moreover, at least in Norway, dam construction projects were often coupled with positivistic stocking programs as mitigation and replacement for lost breeding potentials as part of the concession^9^.

Different hatchery breeding strategies likely have different impacts on the genetic composition of released fish. For example, rearing repeatedly from a single captive broodstock without replacing individuals, referred to in this paper as traditional multi-generational supportive breeding, can result in inbreeding patterns and genetic drift because the same individuals are crossed and their offspring released to the wild where they can breed with each other^10^. By contrast, modern single-generational supportive breeding, with annual replacement of the parental fish with wild-caught individuals that are used only once for breeding purposes (i.e., one generation), is expected to have fewer negative impacts because the number of breeders should theoretically be larger and therefore, genetic diversity should be better preserved. Furthermore, the fact that the fish are not as long in captivity should reduce domestication effects and selection for hatchery conditions, which in turn may lead to lower survival and reduced fitness of released fish in the wild^11,12^.

### Brown trout in the subarctic Pasvik River in northern Norway

**Figure 1:**
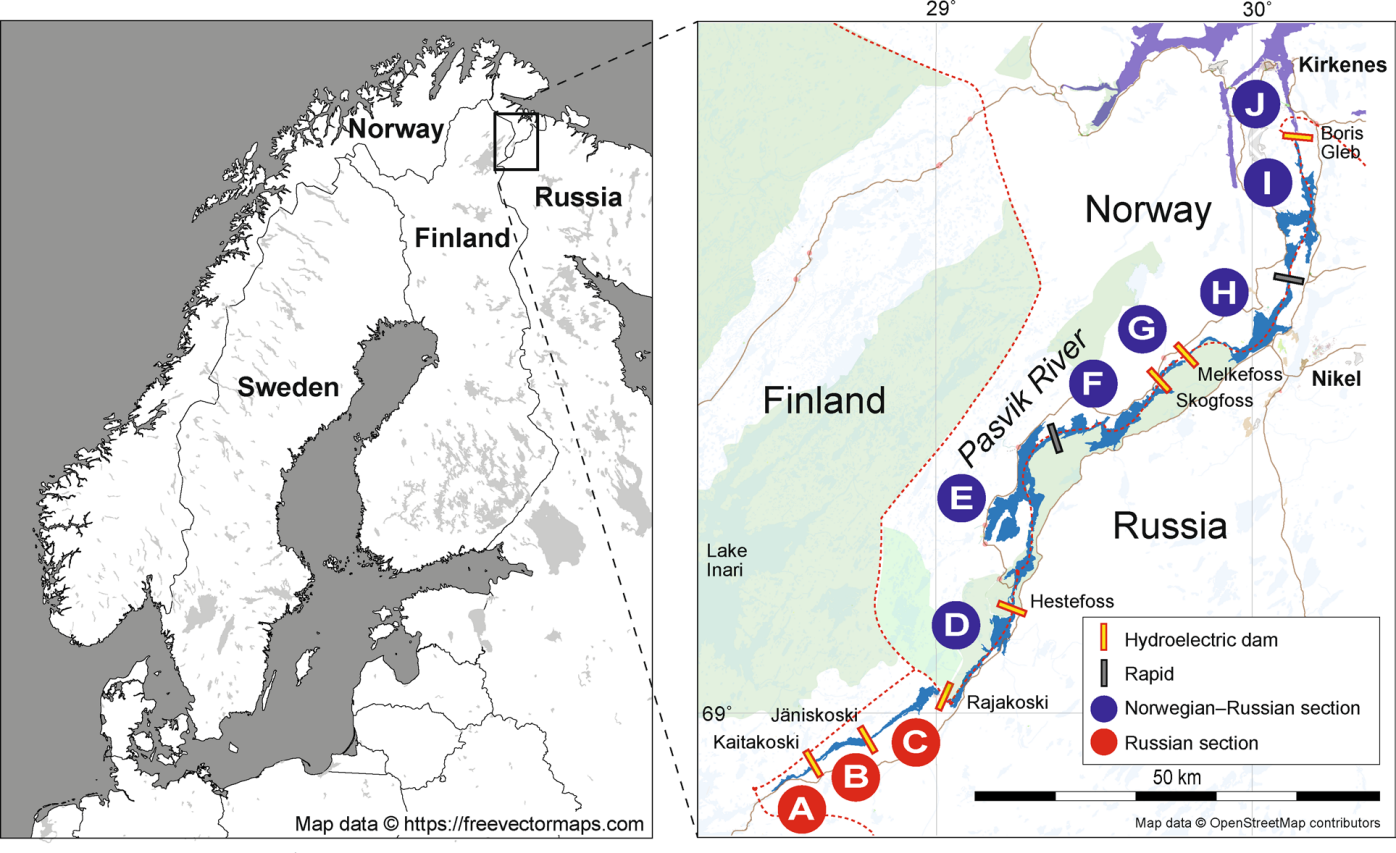
Overview of study area. The different river parts that are separated by hydroelectric dams (orange) are denoted by different letters (A–J). This is a modified map from^4^ based on an OpenStreetMap template (https://www.openstreetmap.org/#map=7/69.701/24.053), which is available as open data (https://www.openstreetmap.org/copyright).

The Pasvik River is a transnational subarctic river system (Fig. 1) known for its large-growing brown trout (*Salmo trutta* L. 1758). As shown by^4^, the natural genetic integrity of the population is threatened by habitat destruction, fragmentation caused by hydropower developments, and the long-term practice of supportive stocking. Different management practices in Norway and Russia have led to distinct genetic diversity patterns in this population. Mainly, river zones situated in Russia are not stocked, whereas Norwegian-Russian zones have been stocked with ~ 5000 offspring of local specimens annually since the 1970s to supplement natural recruitment^13,14^. Stocking was introduced because seven hydroelectric dams without fish passes were built between 1932 and 1978. The breeding program was initiated as a requirement for building the last dam (i.e., Melkefoss, Fig. 1). The dams fragmented the river habitat and destroyed natural spawning and nursery grounds, reducing brown trout recruitment potential, and creating barriers to migration and gene flow among river zones. The Norwegian-Russian river zones nowadays contain an estimated 70–90% stocked fish^15–17^. Furthermore, some of these stocked river zones show signs of recent bottlenecks, suggesting that they are losing genetic variation^4^.

The wild large-growing brown trout population in this watercourse may be facing two major challenges concerning the preservation of genetic integrity: reduced effective population size and potential mating with stocked fish^18,19^. Both factors can lead to allele frequency shifts and losses along with loss of local population structure. Different management and breeding practices can also manifest themselves in morphological changes with different outcomes. Generally, the rate of morphological change introduced by human activity outpaces natural causes^20,21^. In fish species, multigenerational breeding of the same broodstock may lead to stronger deviations from the wild phenotype than single-year breeding programs because of domestication effects^22^, potentially including rapid evolutionary effects^23^. Further, breeding of closely related specimens, either due to a small start population or interbreeding of generations, may lead to inbreeding depression, for instance, for adult size and weight^24^. Inbreeding depression can negatively affect long-term survival of populations and thereby compromises supportive breeding programs.

To test this, we applied a temporal study design to estimate genetic and demographic changes during 17 years of stocking (2001–2018). Hypotheses included declining genetic diversity and/or allele frequency changes as well as bottlenecks and reductions in effective population sizes through time due to increasing stocking impacts (i.e., homogenization effect and/or replacement). Furthermore, we investigated the differential effects of traditional and modern supportive breeding practices, which shifted in the mid-1990s from a multi- to a single-generational (annually renewed) broodstock of local trout specimens. Overall, the aim was to study how different breeding practices have influenced genetic variation and demographics through time in brown trout.

## Methods

### Sample collection

In total, the data set consisted of 593 tissue samples of brown trout from the Pasvik River. From two zones we had time series data including 303 historical samples collected in 2001 and 2007–08 from river zones E and I (Fig. 1, Table 1; referred to as populations E2001, E2007, E2008, and I2007) and 290 contemporary samples collected in 2017–18, of which 175 were from Klütsch et al. (2019)^4^ (DRYAD: https://doi.org/10.5061/dryad.45482t5). For the *contemporary* samples, we only give the zone designation, as shown in Fig. 1 (e.g., A, B, C, etc.) while for *historical* samples, we additionally indicate the collection year as mentioned above. The study was performed in strict accordance with Norwegian legislation. Fish were euthanized by means of cerebral concussion prior to sample collection. A fishing license is required from the fishing right owner. Accordingly, we obtained fishing permissions for the Pasvik watercourse from the County Governor of Finnmark with legal authority through LOV 1992-05-15 nr 47, §13. No ethical permission is required from the Norwegian Animal Research Authority for collection with gill nets and the associated sacrifice of fish (FOR 1996-01-15 nr 23, Norwegian Ministry of Agriculture and Food). Additional samples were obtained by opportunistic collection of samples from local anglers with permission to fish in the river for personal sustenance.

**Table 1:** Genetic summary statistics of river zones and temporal samples.

	N	H_O_ (SE)	H_E_ (SE)	F_IS_ (SE)	A_R_ (SE)	A_PR_ (SE)	A_RG_ (SE)	A_PRG_ (SE)	*r*_*w*_
B	53	0.583 (0.052)	0.589 (0.054)	0.006 (0.023)	3.614 (0.376)	0.165 (0.061)	6.042 (0.894)	0.498 (0.191)	0.069
C	32	0.565 (0.048)	0.606 (0.053)	0.066 (0.022)	3.746 (0.379)	0.148 (0.050)			0.005
E2008	13	0.609 (0.062)	0.624 (0.051)	0.061 (0.056)	3.875 (0.368)	0.058 (0.020)	5.998 (0.877)	0.147 (0.047)	−0.015
E2007	33	0.610 (0.039)	0.638 (0.042)	0.036 (0.026)	3.789 (0.360)	0.071 (0.019)			0.001
E2001A	30	0.582 (0.069)	0.503 (0.051)	−0.141 (0.050)	2.814 (0.211)	0.057 (0.038)	4.538 (0.597)	0.057 (0.03)	0.340
E2001B	22	0.580 (0.073)	0.520 (0.058)	−0.089 (0.037)	2.842 (0.275)	0.053 (0.037)			0.306
E2001C	37	0.647 (0.063)	0.506 (0.049)	−0.269 (0.042)	2.742 (0.233)	0.009 (0.007)			0.379
E2001D	54	0.678 (0.069)	0.529 (0.042)	−0.255 (0.059)	2.608 (0.164)	0.016 (0.013)			0.442
E2001E	44	0.691 (0.054)	0.633 (0.048)	−0.089 (0.024)	3.587 (0.319)	0.042 (0.024)	6.005 (0.873)	0.311 (0.107)	0.122
E2001F	27	0.642 (0.057)	0.651 (0.048)	0.027 (0.033)	4.010 (0.406)	0.187 (0.064)			−0.022
E	23	0.622 (0.053)	0.625 (0.043)	0.018 (0.035)	3.795 (0.362)	0.140 (0.051)	6.224 (0.924)	0.419 (0.120)	0.028
F	25	0.582 (0.055)	0.635 (0.044)	0.104 (0.047)	3.829 (0.361)	0.206 (0.068)			−0.015
G	54	0.633 (0.058)	0.632 (0.053)	0.004 (0.027)	3.801 (0.392)	0.094 (0.025)			0.015
H	51	0.650 (0.047)	0.649 (0.045)	−0.003 (0.029)	3.841 (0.352)	0.109 (0.033)			0.013
I	45	0.640 (0.048)	0.647 (0.049)	0.010 (0.025)	3.812 (0.379)	0.075 (0.029)			−0.002
I2007	43	0.603 (0.044)	0.636 (0.052)	0.035 (0.047)	3.875 (0.382)	0.102 (0.039)	5.905 (0.846)	0.224 (0.113)	−0.015

*N* number of individuals, *H*_*O*_* (SE)*   observed heterozygosity with standard error, *H*_*E*_* (SE)*   expected heterozygosity with standard error, *F*_*IS*_* (SE)*   inbreeding coefficients with standard error, *A*_*R*_  allelic richness, *A*_*PR*_  private allelic richness, *A*_*RG*_  allelic richness per group, *A*_*PRG*_  private allelic richness per group. Standard errors (SE) are given in parentheses. Finally, relatedness estimates per group (***r***_***w***_), based on the Wang estimator, are given.

### Molecular methods

All samples were genotyped at 16 short-tandem repeat (STR) loci and PCR conditions, electrophoresis with an Applied Biosystems 3730xl Genetic Analyzer (Applied Biosystems, UK), and allele scoring are detailed in Supplementary Material S1 and Klütsch et al.^4^.

### Genetic variation

Temporal replicates were treated as separate groups in all analyses unless stated otherwise. MICRO-CHECKER 2.2.3^25^ was used to detect possible genotyping errors, large allele dropouts, and null alleles. Deviations from expected Hardy–Weinberg (HW) proportions were tested with the software GENEPOP 4.7.2^26^ using the Markov chain method with 10,000 dememorization steps, 5000 batches, and 10,000 iterations to estimate exact P-values. The same settings were employed to test for linkage disequilibrium between pairs of loci. Standard summary statistics, including allelic proportions per STR locus for the time series data, were calculated with GenAlEx 6.51b2^27,28^. ADZE 1.0^29^ was used to estimate allelic richness (A_R_) and private allelic richness (A_RP_) corrected for differences in sample sizes when considering the different zones separately (standardized sample size of 10) and at the group level based on genetic clusters, stocking history, and different time periods (standardized sample size of 50). For the second analysis, six major groups were therefore identified: (i) the Russian zones B–C as no supportive breeding occurs there, (ii) E2008/2007 because these samples are from an intermediate time period, (iii) E2001A-E2001D as representative of the traditional breeding practice (i.e., multigenerational), (iv) E2001E and E2001F as these represent the modern stock and the stock that most closely resembles wild trout in the river system (we did not separate these two as some admixture occurs between them), (v) zones E–I as these zones are regularly stocked, and (vi) I2007 because these samples represent an intermediate time period.

### Relatedness

Since stocking is typically performed with offspring from a small subset of individuals, it is expected that stocked fish show closer family relationships. In addition, in salmonid species, the juveniles are often found in shoals/schools of related individuals^30^ and it has been shown that detection of genetic clusters in STRUCTURE^31^ and DAPC^32^ can be caused by groups of closely related individuals in the data set^30^. Although we only sampled adult fish 27–70 cm in length, encompassing several age classes, we estimated relatedness based on allele frequencies with the R package RELATED v.1.0^33^ to test whether a higher-than-expected relatedness level is present in any of the groups studied. First, we used the *compareestimator* function to compare the performance of four different relatedness estimators. The most fitting relatedness estimator based on the highest correlation coefficient (*R*) was used to estimate relatedness within spatial and temporal groups and to estimate pairwise relatedness within those groups that showed higher-than-expected relatedness.

### Demography

We used the program BOTTLENECK 1.2.02^34^ to assess whether recent genetic bottlenecks, linked to breeding practices over the last 20–30 years, could be found. The underlying algorithm assumes that allelic diversity is lost faster than heterozygosity. Hence, it tests for heterozygosity excess compared to expectations at mutation-drift equilibrium^35^. We tested two mutation models, the infinite-alleles-model (IAM) and the two-phase-model (TPM) following the recommendations by^36^. The latter encompasses two mutation models, the IAM and the stepwise mutation model (SMM), and different proportions of STRs can display either one of these mutation models. The TPM model was run three times for each population with the percentage of stepwise mutations being 20%, 50%, and 70%, respectively. We used the 1-way Wilcoxon sign-rank test^37^ and 10,000 iterations to determine significance.

### Effective population size

The effective population size may decrease because many individuals from the same parents are released relative to the natural recruitment in the river. To test for differences of effective population size in a spatio-temporal context as well as investigate the effects of different breeding practices (i.e., multi- *versus* single-generational), we used the program NeEstimator 2.1^38^. We calculated the effective population size (Ne) with three point estimators (i.e., Linkage Disequilibrium method (LD;^39^), Heterozygote Excess method (HE;^40^, and the Molecular Coancestry method (MCA;^41^). In all instances, we used allele frequencies ≥ 0.02 as recommended by^38^. We obtained confidence intervals with the jackknife method of^39^. We added the sibship frequency (SF) estimator as a fourth effective population size estimator and used COLONY 2.0.6.5^42^ with default priors, because^43^ showed through simulations that the most widely used LD method overestimates Ne when sample sizes are considerably smaller than the actual Ne, and that it can be inaccurate when the assumption of random mating is violated.

### Population-genetic differentiation

Pairwise population genetic differentiation was estimated with G_ST_^44^ and Jost’s D^45^ using GENALEX 6.51b2 with 9,999 random permutations^27,28^. To correct for multiple testing, the false discovery rate as described by Benjamini and Hochberg (^46^—BH-FDR) was calculated with the *p.adjust* function in R version 3.6.0^47^ to minimize Type I and II errors, while controlling FDR by using an ɑ-level of 0.01^48^.

STRUCTURE 2.3.4^31^ was run with the admixture model with correlated allele frequencies^49^ for several datasets. An initial run included all samples from the entire river and all time periods. In addition, the two river sections with temporal samples (i.e., zones E and I) were separately analysed to check whether additional substructuring could be found and to confirm the initially identified genetic clusters. Using two sets of runs, one with the LocPrior option and one without, K = 1–10 was tested with 40 replicates each. Run parameters further included 1,000,000 MCMC steps and a burn-in period of 100,000. The LocPrior option allows for detection of weak population-genetic structure^50^. All STRUCTURE analyses were carried out on the CIPRES Portal v3.3 (https://www.phylo.org/;^51^ that allows for parallelised calculation with the R package PARALLELSTRUCTURE^52^. To summarize replicates and determine the most likely number of genetic clusters in the data set, the program STRUCTURESELECTOR^53^ was utilized to account for uneven sample sizes in the data set. Four different estimators introduced by^54^ were calculated: the median of means (MedMeaK), maximum of means (MaxMeaK), median of medians (MedMedK), and maximum of medians (MaxMedK). Summary bar plots were prepared with the program CLUMPAK^55^.

As initial tests indicated that highly related individuals were present in some of the identified genetic clusters, we ran a second set of Bayesian assignment analyses with a reduced data set to account for potential false identification of genetic clusters by the presence of closely related specimens in the data set^56^. This second set of Bayesian assignment analyses had closely related individuals randomly reduced by ≥ 50% following recommendations by^30^, whose simulations showed that total purging of related individuals also leads to biases^30^.

In addition, a Discriminant Analysis of Principal Components (DAPC) was conducted to test whether temporal populations within zones E and I are genetically different using the ADEGENET package^32^ in R version 3.6.0^47^. To avoid over-fitting, a cross‐validation function with 100 replicates was used to determine the optimal number of principal components to be retained. Training *versus* test set proportions were 0.1 and 0.9, respectively.

Finally, a Principal Component Analysis (PCA) was carried out in the ADEGENET package^32^ in R version 3.6.0^47^, which is interfaced with the package ADE^57^. This was done as an additional verification that identified groups in DAPC can also be found without pre-defined groupings.

### Morphological variation

For the historical samples in E2001, measures of body size, weight, and a morphology-based classification into stocked and wild fish were available (Table 2), which were used to test whether genetic clusters showed differences in these morphological traits and whether there was a correlation between genetic diversity and the origin of the fish. The classification into stocked *versus* wild fish was done by the fishermen, based on characteristic fin damage in stocked fish^14^. The accuracy of this classification was evaluated by by otolith analysis^17^ of a large subsample (N = 202), resulting in the same classification as the fin damage method in 97.5% of the cases.

**Table 2:** Overview of length and weight data for the historical samples in zone E, sorted according to genetic subgroups E2001A–F.

Genetic cluster	N_LENGTH_	Mean length in cm (SD)	N_WEIGHT_	Mean weight in kg (SD)	N_CLASSI-FICATION_	% of fish classified as wild (N)	% of fish classified as stocked (N)
E2001A	29	38 (3.1)	27	0.724 (0.16)	30	3.3 (1)	96.7 (29)
E2001B	20	38.8 (1.4)	20	0.759 (0.13)	22	4.5 (1)	95.5 (21)
E2001C	31	38.1 (2.4)	32	0.769 (0.17)	37	8.1 (3)	91.9 (34)
E2001D	50	37.8 (1.9)	50	0.746 (0.13)	54	3.7 (2)	96.3 (52)
E2001E	44	41.8 (4.8)	40	0.894 (0.25)	44	15.9 (7)	84.1 (37)
E2001F	25	45.5 (7.1)	24	1.160 (0.46)	27	63.0 (17)	37.0 (10)

In addition, the morphological classification into wild and stocked fish based on fin damage is given in percent and total numbers. For each measurement or classification, the number of individuals is given (i.e., N_LENGTH_, N_WEIGHT_, N_CLASSIFICATION_).

Initial statistical tests included tests for multicollinearity assessed by a Pearson correlation, homogeneity of variances assessed by Levene’s test^58^, and tests for outliers with the *identify_outliers()* function in the rstatix package^59^. Normality was evaluated by plotting the correlation between the data and a normal distribution per genetic cluster in a QQ plot, because with high sample sizes, the typically applied Shapiro–Wilk test becomes sensitive to even minor deviations from normality. Subsequently, we used a Welch one-way ANOVA plus Games-Howell post hoc tests for all pairwise comparisons. P-values were adjusted for multiple testing using the *TukeyHSD()* function in the rstatix package^59^.

## Results

### Genetic variation and summary statistics

Initial tests showed strong substructure within the historical population E2001, which was ultimately sorted into six subpopulations (referred to as E2001A to E2001F), based on high assignment scores, to break down linkage disequilibrium. In the final population set, linkage disequilibrium was significant (p < 0.05) in only 7/2051 pairwise comparisons after Bonferroni correction. Deviations from Hardy–Weinberg equilibrium were observed in one locus (SsaD157) in two populations (zone I and I2007) after Bonferroni correction.

Heterozygosity values (Table 1) suggested that historical samples from zone E showed negative F_IS_ estimates. Allelic richness and private allelic richness, as well as allelic proportions per locus were particularly low in the historical genetic clusters, except for E2001E and E2001F, which corresponded to the contemporary populations (Table 1, S15–S30). Four alleles were only found in E2001E and F and not in any other historical or contemporary groups.

MICRO-CHECKER identified only one locus (SsaD157) in one group (I2007), showing a significant presence of null alleles, and found no signs of large allele dropout at any locus. Furthermore, only one population (zone E) showed potential scoring errors due to stutter at one locus (OMM1152). Hence, all loci were retained for further analysis.

### Tests for genetic clustering and differentiation

**Figure 2:**
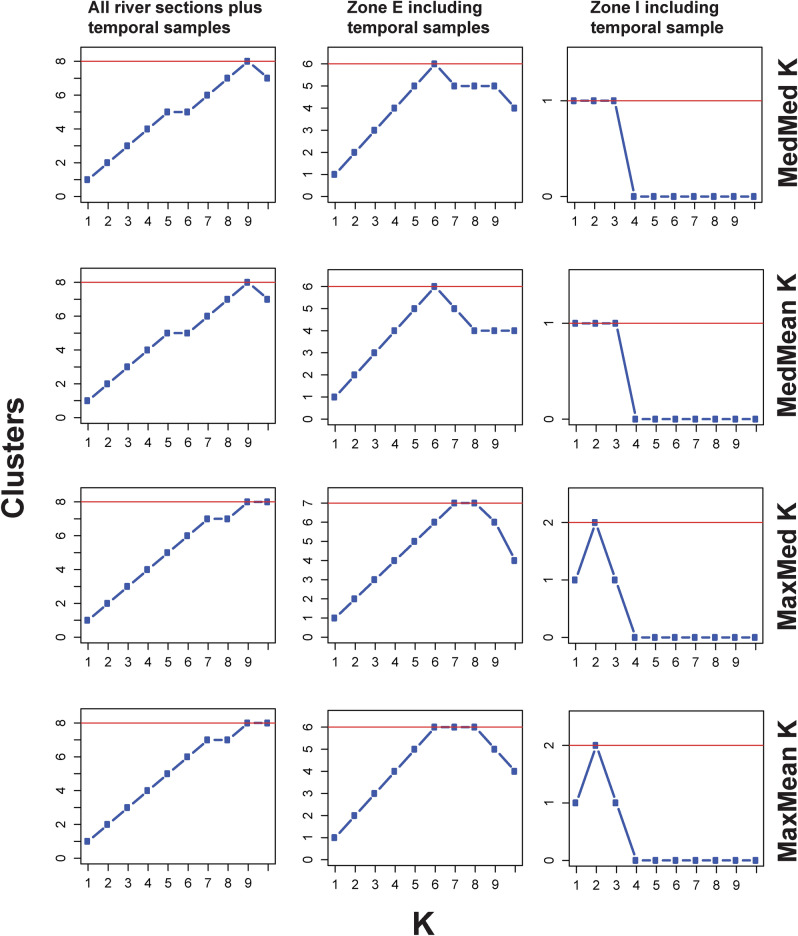
STRUCTURESELECTOR results. Here, we show only runs without use of the LOCPRIOR option; runs for which the LOCPRIOR option was used can be found in the supplementary material. Left panel: STRUCTURESELECTOR results for the entire data set (i.e., all main river zones plus temporal samples), middle panel: STRUCTURESELECTOR results for zone E, right panel: STRUCTURESELECTOR results for zone I. The four different estimators introduced by^54^ are listed on the right-hand side. The most likely number of genetic clusters is indicated by a red line.

**Figure 3:**
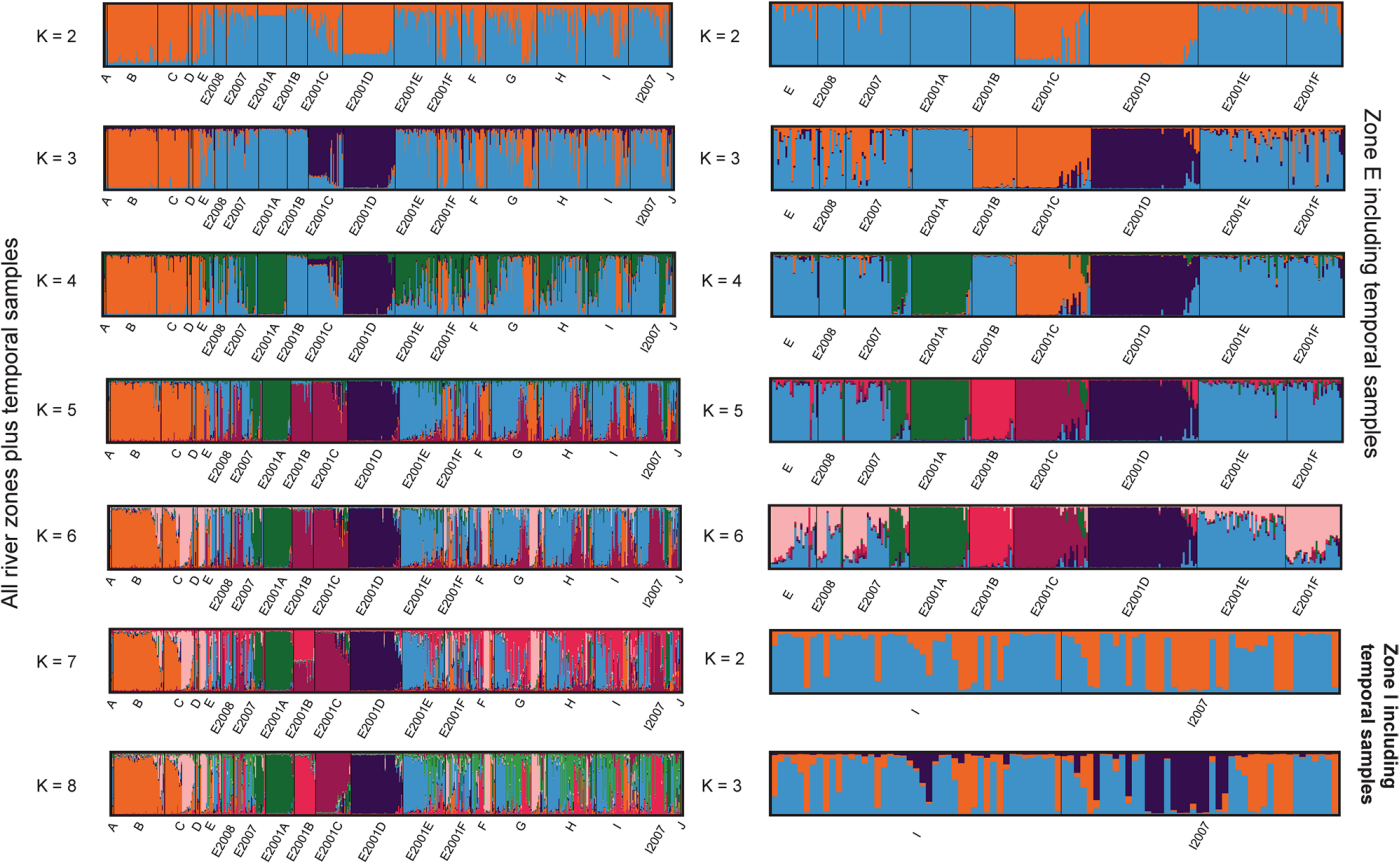
Bar plots for the different STRUCTURE runs. Here, we show only runs without use of the LOCPRIOR option; runs for which the LOCPRIOR option has been used can be found in the supplementary material. On the left-hand side, STRUCTURE bar plots for all investigated river zones (i.e., A–J) plus temporal groups (i.e., E2008, E2007, E2001A-E2001F, and I2007), are shown. On the right-hand side, additional STRUCTURE bar plots for zone E and I are displayed that include temporal samples.

For all samples combined, STRUCTURE and STRUCTURESELECTOR determined K = 8 as the likely number of genetic clusters (Figs. 2 and 3). Spatially, genetic differentiation occurred between contemporary non-stocked and stocked river zones, consistent with^4^ (Fig. 3). Temporally, elevated genetic structure occurred in historical compared to contemporary samples (Figs. 2 and 3), with three out of six genetic clusters found only among historical samples (i.e., E2001A, C, and D) (Fig. 3). Analysing only river zones with both historical and contemporary samples (i.e. zones E and I) confirmed these results, providing evidence that three genetic clusters went extinct during the 17-year study period (Fig. 3). For zone I, the historical sample was weakly genetically differentiated from the contemporary sample (Fig. 3).

**Figure 4:**
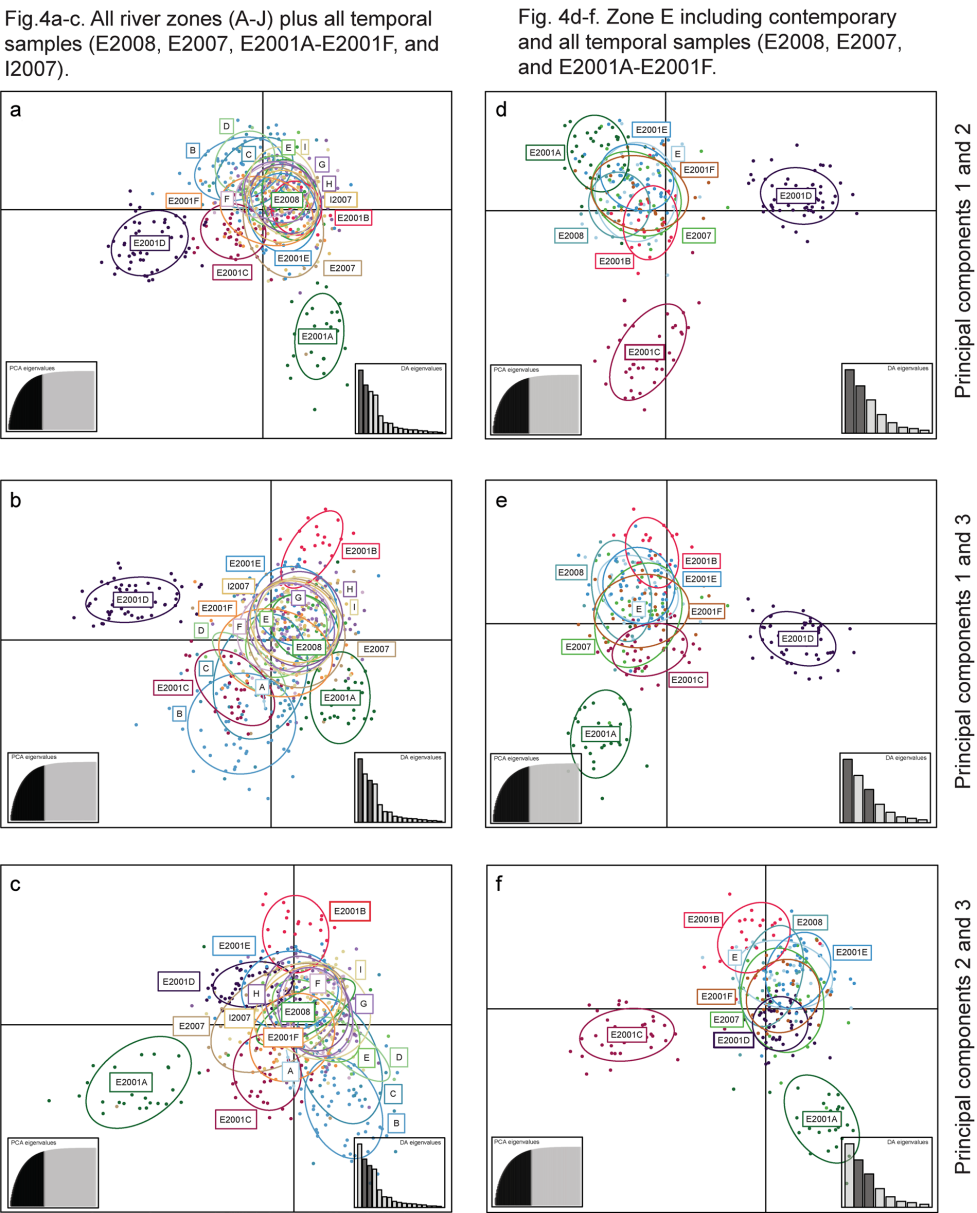
Discriminant analysis of principal components (DAPC) scatter plots based on 16 STR-markers and all individuals for brown trout (*Salmo trutta*). **(a–c)** DAPC scatter plots for all investigated river zones (i.e., A–J) plus temporal groups (i.e., E2008, E2007, E2001A–E2001F, and I2007). **(d–f)** show DAPC plots for zone E that include temporal samples. Three DAPC plots for different principal components are displayed.

**Figure 5:**
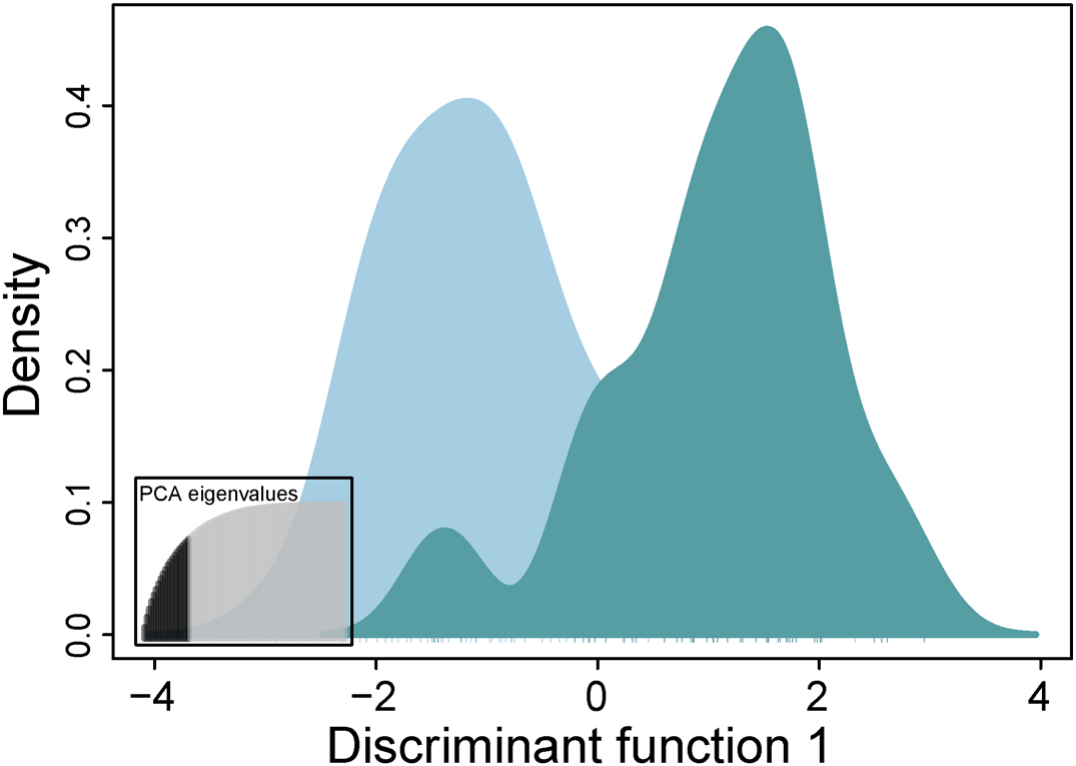
Discriminant analysis of principal components (DAPC) scatter plots based on 16 STR-markers and all individuals for brown trout (*Salmo trutta*) in zone I, including the temporal sample I2007.

The DAPC analysis largely confirmed the STRUCTURE results (Figs. 4a-f, 5), identifying three clearly differentiated historical populations (i.e., E2001A, E2001C, and E2001D; Fig. 4). Also, E2001B was differentiated but showed some overlap with both E2001E and E2001F (Fig. 4d–f). Analysing zone E groups alone, E2001A-D were genetically differentiated from both more recent historical samples E2007/E2008 and contemporary samples (Fig. 4d–f), whereas E2001E-F clustered with contemporary populations. In zone I, the historical sample I2007 was genetically different from the contemporary sample (Fig. 5). Re-running the analyses with the reduced data set did not change the results, confirming that the observed patterns were not caused by high relatedness in some groups (Supplementary Material Fig. S1–S8).

The results of the PCA confirmed the DAPC results (Supplementary Material Fig. S9–S14), although distances between groups were smaller, as expected.

Estimates of population-genetic differentiation using G_ST_ and D_EST_ were largely significant in pairwise assessments (Supplementary Material Tables S1 and S2). Historical (stocked) genetic clusters E2001A–D were genetically differentiated from the contemporary stocked populations. In comparison, the historical groups E2001E–F showed relatively low genetic differentiation both from the more recent historical samples E2007/E2008/I2007 and most of the contemporary populations. Finally, the historical sample from I2007 was weakly but significantly differentiated from the contemporary sample in the same zone.

### Relatedness, bottlenecks, and effective population size

For our data set, the relatedness estimator (rw) by^60^ showed the highest correlation coefficient between observed and expected values (r = 0.839). Historical samples E2001A–D (Table 1) displayed considerably higher-than-expected relatedness estimates. Randomly removing 50% of the closely related individuals and re-analysis in STRUCTURE and ADEGENET led to no loss of population structure (Supplementary Material Tables S1 and S2, Supplementary Material Figures S5–S8).

Both bottleneck tests (Table 3) and estimates of effective population size (Table 4) pointed to reduced genetic variation and effective population sizes in the historical genetic clusters that went extinct (E2001A–D), compared to those that survived (E2001E–F). Of those that survived, E2007F showed no signs of bottlenecks and had the largest effective population size, whereas E2001E showed signs of a recent bottleneck and an intermediate effective population size. The results also confirmed previous findings^4^ that non-stocked zones in Russia (i.e., B and C) do not show signs of demographic bottlenecks while stocked zones G, H, and I showed consistent signs of bottlenecks (Table 3). Regarding effective population sizes, the non-stocked Russian zones B and C showed either comparable (SF estimator) or higher estimates (LD estimator) than the stocked Norwegian-Russian zones E–I (Table 4).

**Table 3:** Tests for genetic bottlenecks using BOTTLENECK.

	IAM	TPM_70	TPM_50	TPM_20
B	**0.011**	0.490	0.174	0.080
C	**0.007**	0.334	0.161	**0.047**
E	**0.022**	0.470	0.264	0.161
E2008	**0.001**	**0.007**	**0.003**	**0.001**
E2007	**0.012**	0.217	0.106	**0.042**
E2001A	**0.012**	0.106	0.058	**0.042**
E2001B	**0.000**	**0.004**	**0.001**	**0.000**
E2001C	**0.005**	0.096	**0.042**	**0.017**
E2001D	**0.000**	**0.000**	**0.000**	**0.000**
E2001E	**0.000**	**0.001**	**0.000**	**0.000**
E2001F	**0.008**	0.334	0.161	0.072
F	**0.022**	0.298	0.149	0.096
G	**0.007**	0.088	**0.025**	**0.012**
H	**0.007**	0.126	**0.037**	**0.019**
I	**0.003**	**0.012**	**0.007**	**0.004**
I2007	**0.004**	0.052	**0.015**	**0.009**

Tests were performed using the infinite-allele model and the two-phase mutation model (TPM). For the latter, different proportions of STR loci that follow the stepwise mutation model were used (i.e., 70, 50, and 20%, respectively). P-values according to a 1-way Wilcoxon rank test for heterozygote excess are given and significant values are highlighted in bold font.

**Table 4:** Effective population-size estimates for all zones and temporal groups identified.

	LD (95% CI)	HE (95% CI)	MCA (95% CI)	SF estimator (random mating) (95% CI)	SF estimator (non-random mating) (95% CI)
B	101.1 (43.5–56,362.3)	∞	19.6 (1.4–61.0)	45 (30–71)	35 (22–57)
C	151.5 (69.0–∞)	∞	11.4 (2.4–27.6)	34 (21–57)	24 (14–46)
E	157.5 (74.6–∞)	∞ (34.3–∞)	∞	27 (16–50)	21 (12–43)
E2008	58.7 (14.8–∞)	∞ (30.8–∞)	33.9 (0.0–170.3)	16 (8–42)	12 (6–33)
E2007	38.3 (20.1–123.3)	∞	9.4 (6.0–13.6)	28 (16–50)	21 (12–42)
E2001A	4.5 (2.7–7.8)	5.3 (3.4–14.9)	5.8 (2.8–9.8)	3 (2–∞)	3 (2–∞)
E2001A_RED	5.9 (2.9–10.1)	5.2 (3.3–13.6)	11.9 (2.5–28.6)	3 (2–∞)	3 (2–∞)
E2001B	25.7 (8.9–∞)	7.7 (4.0–∞)	13.4 (2.2–34.5)	5 (2–20)	6 (3–20)
E2001B_RED	34.8 (8.7–∞)	10.3 (4.5–∞)	12.6 (3.8–26.6)	11 (6–28)	11 (5–28)
E2001C	7.2 (3.7–11.7)	3.4 (2.6–4.9)	35.3 (0.9–130.3)	3 (2–∞)	3 (2–12)
E2001C_RED	7.3 (3.1–13.7)	3.5 (2.7–5.2)	∞ (∞)	4 (2–12)	4 (2–12)
E2001D	7.8 (3.1–16.8)	2.6 (1.9–4.2)	8.0 (3.2–15.0)	3 (2–∞)	3 (2–12)
E2001D_RED	6.1 (2.9–12.8)	2.6 (1.9–4.1)	13.4 (2.2–34.5)	3 (2–∞)	3 (2–12)
E2001E	27.7 (18.4–45.2)	16.2 (6.8–∞)	23.3 (2.8–64.8)	23 (13–43)	23 (14–42)
E2001F	62.2 (28.4–1588.0)	∞ (22.6–∞)	624.8 (0.6–3136.4)	34 (20–60)	28 (16–52)
F	60.1 (33.2–198.5)	∞	13.3 (4.3–27.3)	31 (18–57)	22 (12–43)
G	63.9 (42.1–113.1)	∞ (18.1–∞)	7.4 (3.5–12.7)	54 (36–85)	50 (32–75)
H	97.7 (58.6–229.0)	∞ (83.3–∞)	10.3 (5.3–16.9)	42 (27–67)	35 (22–57)
I	123.7 (55.8–336,383.2)	∞	18.4 (8.6–31.7)	45 (29–73)	36 (22–59)
I2007	23.9 (15.3–40.6)	∞	∞	35 (22–58)	27 (17–50)

We used four different estimators: linkage disequilibrium method (LD^38,39^), heterozygote excess method (HE^40^, molecular coancestry method (MCA^41^), and the sibship frequency estimator (SF^43^). For all estimators, the point estimate as well as 95% confidence intervals (CI) are given. In addition, we provide the effective population size estimates for the groups that had 50% of closely related individuals removed to check whether this has any considerable effect on these estimates; these estimates are labelled with the suffix _R.

### Morphological data

**Figure 6:**
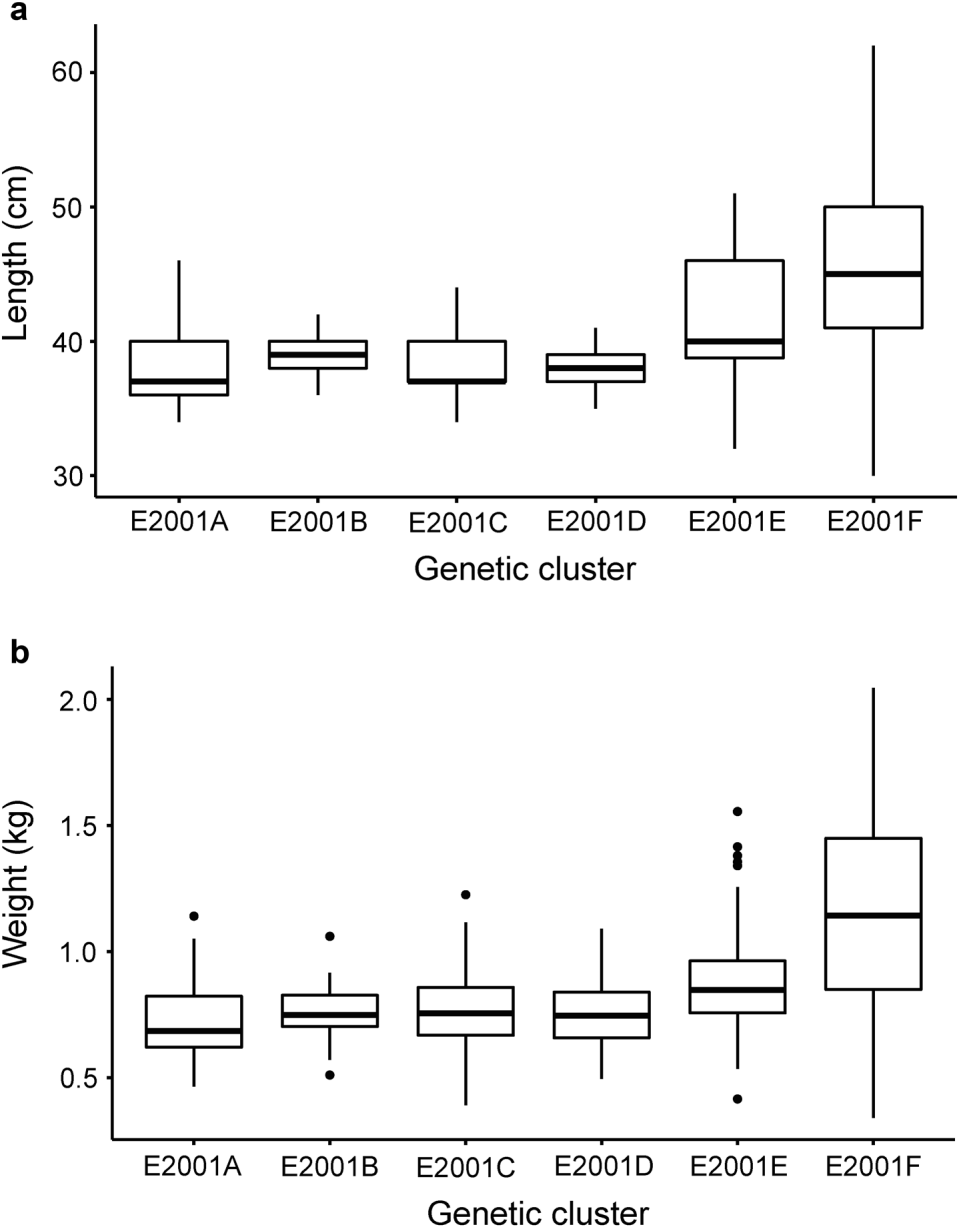
Box plots showing **(a)** length distribution, and **(b)** weight distribution in historical groups E2001A–E2001F. Black line in boxes indicates the mean.

Exploratory statistical tests showed strong multicollinearity between the variables weight and length (r = 0.9, p < 0.0001), but also differences in variances among the groups defined based on genetics (p < 0.0001). Therefore, we used a Welch one-way ANOVA test that does not assume homogeneity of variances in combination with a Games-Howell post hoc test for each variable separately. Prior to the analyses, outliers were removed from the data sets; however, for weight, after the first round of outlier removal, more outliers were found that remained in the analysis. For both length and weight, the ANOVA was significant (length: p < 0.0001; weight: p < 0.0001). For length, the pairwise post hoc tests showed that E2001E and E2001F were significantly different from all other genetic clusters, but not from each other (Fig. 6a). For weight, a similar result was found, but E2001E was not significantly different from E2001B and E2001C (Fig. 6b). Genetic clusters E2001A, E2001B, E2001C, and E2001D did not significantly differ in length and weight from one another. Regarding the morphological classification of caught fish into wild and stocked, E2001F consisted of 63% wild fish, followed by E2001E with ~ 16% wild fish while genetic clusters E2001A-D showed very low proportions of wild fish (Table 2).

## Discussion

Our results revealed that significant temporal changes in genetic structure, effective population size, and allelic composition occurred in the trout population of the Pasvik River during 17 years (i.e., 2001–2018). Three unique genetic clusters could only be found in historical samples within zone E, likely being the result of traditional breeding practices leading to considerable allele frequency shifts. A fourth genetic cluster became rare with time, although still present in low numbers and as admixed individuals at the end of the 17-year study period. In all these four genetic clusters that either disappeared or declined during the study period, genetic diversity measures and effective population sizes were distinctly lower than in the modern breeding stock and the wild population, which was consistent with indications of recent bottlenecks and considerably lower effective population sizes. Overall, these results suggest a loss of genetic diversity and a homogenization effect as well as isolation by time.

Interbreeding between the historical groups was marginal, except for 2001E and F. Previous studies in salmonids found similarly low introgression levels from domestic fish into the native population^61,62^. Potential causes include both lower rates of survival and reproduction and changes in the timing of maturity and spawning introduced by captive-breeding programs^11,63–67^. Shifted spawning timing may also contribute to non-random mating or reproductive isolation by time (temporal assortative mating;^68^), resulting in low admixture with wild populations^64^. If new parents are caught every year for breeding, these effects may be less severe, but they are still present^11,65^. For example, in a meta-analysis, relative reproductive success of early-generation hatchery-reared salmonids in the wild was only 50% on average compared to wild fish^65^. Importantly, genetic effects seem to be present even in F2 generation specimens that have lived their entire lives in natural environments^11^. Further, mortality rates may be higher in captivity-bred than wild fish because of less efficient foraging behaviour, leading to smaller body sizes and poor physical condition, and more risk-prone or exploratory behaviour that may result in higher predation and angling pressure^67^. The progeny of hatchery-raised trout may potentially also suffer from fewer resources at egg hatching in the wild, as early juvenile survival is positively correlated to egg size. Egg size typically increases with mother size and may be smaller than in wild fish for two reasons; (1) there may be a hatchery selection towards smaller egg size^23^, and (2) hatchery fish grow less than wild fish. The latter will also reduce the mothers’ ability to make deep and well-protected nests^69^. These factors may select particularly against domestic trout that grew up in artificial environments and have reduced fitness in comparison to wild trout^11^, but also be detrimental to wild fish if the characters are introgressed back to the wild populations.

Introgression between domestic and native trout can be density-dependent, increasing with the relative size of the domestic population^8^. Although this may partly explain the resilience of the native group with the highest proportion of wild fish (E2001F), it does not explain why domestic fish did not interbreed. Christie et al.^10^ pointed out that there is a trade-off between the number of stocked fish that should be allowed on the spawning grounds because releasing very few might not help the goal of supportive breeding whereas allowing too many will reduce the effective population size considerably.

There were no obvious geographical barriers that can explain the strong separation of genetic clusters within zone E. The relaxation of linkage disequilibrium observed when sorting into different groups and the elevated relatedness of individuals in four out of six groups (i.e., E2001A–D), suggest non-random mating. Ecological, behavioural, historical, and anthropogenic factors may explain genetic sub-structuring of fish populations^70^. We can exclude the possibility of food partitioning between juveniles and adults as only adults were collected. Furthermore, as coregonids (mainly vendace *Coregonus albula*) are the main prey, constituting > 90% of the diet of adult brown trout in the study system^14,15^, we can also exclude the possibility of trophic polymorphism as a mechanism for population sub-structuring.

Our results are consistent with these studies, given that the four genetic clusters E2001A–E2001D represent domestic multi-generational broodstocks. The clear genetic separation and low admixture rates of the genetic clusters in combination with the phenotypic classification of being stocked fish support this. Further, the high relatedness and low allelic richness give further credence to this interpretation. Differences in morphological traits, like body size and weight, between domestic and wild fish have also been found to be linked to hatchery-breeding^63^. However, size variation within those four groups suggests the presence of multiple generations. Thus, either a low reproduction rate was present, or several age classes were released; however, the latter seemed to have been stopped sometime in the 1980s (Pasvik Kraft, personal communication). In any case, reproductive success, particularly interbreeding with wild trout, was low. Also, stocked fish from groups E2001A–D did not interbreed with each other, suggesting that all released stocks had very low rates of survival and reproductive success.

Our results suggest that particularly traditional stocking practices can lead to severe and rapid reductions in effective population sizes and allelic diversity, strong linkage disequilibrium, and increased relatedness in multiple-year breeding programs. Small numbers of breeders plus variation in reproductive success contribute to this pattern. This is consistent with findings by others (e.g.,^10^). The most likely explanation for the four observed genetic clusters is that they correspond to four brood years (i.e., four different instances of releases of hatchery-bred fish) with very low reproduction levels in the wild. These traditional breeding practices have also led to significantly smaller body sizes/weight than found in wild fish. Together with the common signatures of recent bottlenecks and low genetic diversity, inbreeding depression was a likely factor in the traditional breeding program.

The results indicated that the change to supportive breeding practices, using new parental fish every year to reduce the number of crossings per parent, has led to a ‘modern’ stocked group (i.e., E2001E), which showed higher allelic richness and effective population sizes than the previously stocked groups and which co-exists and interbreeds with wild brown trout in the river. Nevertheless, E2001F, the group that most closely resembled wild brown trout at the time, harboured private alleles that were not found in any modern samples, suggesting that genetic diversity was lost through time. Similarly, bottlenecked modern river zones indicate an ongoing loss of genetic diversity^4^. In addition, also population E2001E, suggestively representing the modern breeding practices, showed signs of bottlenecks. Thus, the effective size of the modern breeding stock is larger than in historical practices, but it is not large enough to prevent further loss of genetic diversity.

One remaining potential problem in this system is that annual catches for the broodstock come from the same zone (i.e., H) and that due to the dams, natural migration is limited or non-existent. This probably results in a situation in which the local fish population encompasses a high proportion of stocked fish, as shown by the current study and others^15,16^. Using the Bayesian assignment scores and under the assumption that the Russian non-stocked zones are genetically representative of wild fish, the estimated proportions of wild fish in the contemporary stocked Norwegian-Russian zones equalled 47.8% (zone E), 32.0% (zone F), 13.0% (zone G), 3.9% (zone H), and 8.9% (zone I). This gives an average of 16.2% of wild fish in the stocked zones, but with considerable variation between zones. For example, zone H showed the lowest proportion of wild fish; however, this is the zone in which breeding specimens are caught annually. To preserve natural genetic diversity, breeding practices will need to be refined to increase the number of unrelated breeders and the proportion of wild specimens in the breeding program. Four out of the six zone E populations (E2001B–E) showed largely consistent significant bottlenecks. The main currently surviving wild population in zone E (i.e., E2001F) showed neither elevated relatedness estimates and nor genetic bottlenecks. The other population (E2001E) corresponded to the main contemporary population of the Norwegian-Russian part of the river where stocking and genetic bottlenecks are common^4^. Several factors may explain these results. The bottlenecked historical populations from E2001B-D could already back then have suffered from considerable population decline and ultimately gone partially extinct, whereas the still occurring contemporary population E2001E may be impacted by stocking practices. Stocking and destruction of spawning habitats occurred also before 2001 and may have led to population declines. The only other surviving genetic cluster E2001F shows partially admixed ancestry with E2001E and this may explain the non-significant bottleneck tests. Effective population sizes in non-stocked Russian zones seem to be comparable to or higher than in stocked Norwegian-Russian zones. Thus, stocking does not appear to increase effective population sizes in any significant way, and in worst case scenarios, decreases them considerably^10^.

In conclusion, our study documents large impacts on the genetic structure and genetic diversity of brown trout populations from annual compensatory stockings following construction of hydropower dams. Four genetic clusters were morphologically identified as stocked fish and showed consistently lower genetic diversity, higher within-group relatedness, lower effective population sizes, and significantly smaller body sizes than the other two genetic clusters, which were genetically more similar to contemporary stocked and wild fish. Over a period of 17 years, three of these genetic clusters within one of the studied river sections went extinct and a fourth cluster distinctly declined, indicating that reproduction success of these groups was low. A change in breeding practices from multi- to single-generational breeding of the locally caught broodstock resulted in the maintenance of higher genetic diversity, but even these modernized breeding practices are not enough to prevent genetic diversity loss when compared to wild fish (see also^4^). Hence, conservation management should carefully assess future breeding protocols to preserve remaining natural genetic diversity in brown trout in the Pasvik River and other rivers where compensatory stockings are implemented. Morphological and molecular screening including the determination of relatedness levels before breeding may be a viable option for quickly implementable conservation measures in this system (e.g.,^71^). However, this cannot replace other efforts like habitat restoration to increase natural reproduction. Alternatives include the abandonment of the breeding program altogether. In many cases this would be very drastic, and potentially detrimental to the fish population and human interest as well if not followed up by other measures. We strongly advise starting by improving conditions for natural reproduction, including increased connectivity by opening fish passages around hydropower installations to facilitate fish migration. Our findings have important implications for stocking program management and indicate that a detailed evaluation will be needed to find the best way forward for stocking practices where such programs exist.

## Supplementary Information

Supplementary Information.

## Data Availability

Additional genetic data generated for this manuscript has been deposited on DRYAD (10.5061/dryad.hx3ffbgf7).

## References

[1] Mimura M (2017). Understanding and monitoring the consequences of human impacts on intraspecific variation. Evol. Appl..

[2] Leigh DM, Hendry AP, Vázquez-Domínguez E, Friesen VL (2019). Estimated six per cent loss of genetic variation in wild populations since the industrial revolution. Evol. Appl..

[3] Habel JC, Husemann M, Finger A, Danley PD, Zachos FE (2014). The relevance of time series in molecular ecology and conservation biology. Biol. Rev..

[4] Klütsch CFC (2019). Genetic changes caused by restocking and hydroelectric dams in demographically bottlenecked brown trout in a transnational subarctic riverine system. Ecol. Evol..

[5] Hansen MM, Fraser DJ, Meier K, Mensberg K-LD (2009). Sixty years of anthropogenic pressure: A spatio-temporal genetic analysis of brown trout populations subject to stocking and population declines. Mol. Ecol..

[6] Savary R (2017). Stocking activities for the Arctic char in Lake Geneva: Genetic effects in space and time. Ecol. Evol..

[7] Hughes JB, Daily GC, Ehrlich PR (1997). Population diversity: its extent and extinction. Science.

[8] Perrier C, Guyomard R, Bagliniere J-L, Nikolic N, Evanno G (2013). Changes in the genetic structure of Atlantic salmon populations over four decades reveal substantial impacts of stocking and potential resiliency. Ecol. Evol..

[9] Vøllestad LA, Hesthagen T (2001). Stocking of freshwater fish in Norway: management goals and effects. Nordic J. Freshwater Res..

[10] Christie MR, Marine ML, French RA, Waples RS, Blouin MS (2012). Effective size of a wild salmonid population is greatly reduced by hatchery supplementation. Heredity.

[11] Araki H, Cooper B, Blouin MS (2009). Carry-over effect of captive breeding reduces reproductive fitness of wild-born descendants in the wild. Biol. Lett..

[12] O'Sullivan RJ (2020). Captive-bred Atlantic salmon released into the wild have fewer offspring than wild-bred fish and decrease population productivity. Proc. R. Soc. B.

[13] Amundsen P-A (1999). Invasion of vendace *Coregonus albula* in a subarctic watercourse. Biol. Conserv..

[14] Jensen H, Bøhn T, Amundsen P-A, Aspholm PE (2004). Feeding ecology of piscivorous brown trout (*Salmo trutta* L.) in a subarctic watercourse. Ann. Zool. Fenn..

[15] Jensen H (2008). Predation by brown trout (*Salmo trutta*) along a diversifying prey community gradient. Can. J. Fish. Aquat. Sci..

[16] Jensen H (2015). Food consumption rates of piscivorous brown trout (*Salmo trutta*) foraging on contrasting coregonid prey. Fish. Manag. Ecol..

[17] Haugland, Ø. *Langtidsstudie av næringsøkologi og vekst hos storørret i Pasvikvassdraget. Mastergradsoppgave i biologi* (Universitetet i Tromsø, Fakultet for Biovitenskap, fiskeri og økonomi, Institutt for arktisk og marin biologi, 2014).

[18] Gossieaux P, Bernatchez L, Sirois P, Garant D (2019). Impacts of stocking and its intensity on effective population size in Brook Charr (*Salvelinus fontinalis*) populations. Conserv. Genet..

[19] Pinter K, Epifanio J, Unfer G (2019). Release of hatchery-reared brown trout (*Salmo trutta*) as a threat to wild populations? A case study from Austria. Fish. Res..

[20] Wringe BF, Purchase CF, Fleming IA (2016). In search of a “cultured fish phenotype”: A systematic review, meta-analysis and vote-counting analysis. Rev. Fish Biol. Fish..

[21] Gossieaux P (2020). Effects of genetic origin on phenotypic divergence in Brook Trout populations stocked with domestic fish. Ecosphere.

[22] Fleming IA, Jonsson B, Gross MR (1994). Phenotypic divergence of sea-ranched, farmed, and wild salmon. Can. J. Fish. Aquat. Sci..

[23] Heath DD, Heath JW, Bryden CA, Johnson RM, Fox CW (2003). Rapid evolution of egg size in captive salmon. Science.

[24] Naish KA, Seamons TR, Dauer MB, Hauser L, Quinn TP (2013). Relationship between effective population size, inbreeding and adult fitness-related traits in a steelhead (*Oncorhynchus mykiss*) population released in the wild. Mol. Ecol..

[25] Van Oosterhout C, Weetman D, Hutchinson WF (2006). Estimation and adjustment of microsatellite null alleles in nonequilibrium populations. Mol. Ecol. Notes.

[26] Rousset F (2008). Genepop'007: a complete reimplementation of the Genepop software for Windows and Linux. Mol. Ecol. Resour..

[27] Peakall R, Smouse PE (2006). GENALEX 6: Genetic analysis in Excel. Population genetic software for teaching and research. Mol. Ecol. Notes.

[28] Peakall R, Smouse PE (2012). GenAlEx 6.5: Genetic analysis in Excel. Population genetic software for teaching and research-an update. Bioinformatics.

[29] Szpiech ZA, Jacobsson M, Rosenberg NA (2008). ADZE: A rarefaction approach for counting alleles private to combinations of populations. Bioinformatics.

[30] Waples RS, Anderson EC (2017). Purging putative siblings from population genetic data sets: A cautionary view. Mol. Ecol..

[31] Pritchard JK, Stephens M, Donnelly P (2000). Inference of population structure using multilocus genotype data. Genetics.

[32] Jombart T, Devillard S, Balloux F (2010). Discriminant analysis of principal components: A new method for the analysis of genetically structured populations. BMC Genet..

[33] Pew J, Muir PH, Wang J, Frasier TR (2015). Related: An R package for analysing pairwise relatedness from codominant molecular markers. Mol. Ecol. Resour..

[34] Piry S, Luikart G, Cornuet J-M (1999). Bottleneck: A computer program for detecting recent reductions in the effective population size using allele frequency data. J. Heredity.

[35] Cornuet JM, Luikart G (1996). Description and power analysis of two tests for detecting recent population bottlenecks from allele frequency data. Genetics.

[36] Peery MZ (2012). Reliability of genetic bottleneck tests for detecting recent population declines. Mol. Ecol..

[37] Luikart, G. Usefulness of molecular markers for detecting population bottlenecks and monitoring genetic change. *Ph. D. Thesis*. (University of Montana, 1997). 10.1046/j.1365-294x.1998.00414.x 9711862

[38] Do C, Waples RS, Peel D, MacBeth GM, Tillett BJ, Ovenden JR (2014). NEESTIMATOR v2: Re-implementation of software for the estimation of contemporary effective population size (Ne) from genetic data. Mol. Ecol. Resour..

[39] Waples RS, Do C (2008). LDNE: A program for estimating effective population size from data on linkage disequilibrium. Mol. Ecol. Resour..

[40] Zhdanova OL, Pudovkin AI (2008). Nb_HetEx: A program to estimate the effective number of breeders. J. Hered..

[41] Nomura T (2008). Estimation of effective number of breeders from molecular coancestry of single cohort sample. Evol. Appl..

[42] Jones OR, Wang J (2010). COLONY: A program for parentage and sibship inference from multilocus genotype data. Mol. Ecol. Resour..

[43] Wang JA (2016). comparison of single-sample estimators of effective population sizes from genetic data. Mol. Ecol..

[44] Nei M, Chesser RK (1983). Estimation of fixation indexes and gene diversities. Ann. Hum. Genet..

[45] Jost L (2008). Gst and its relatives do not measure differentiation. Mol. Ecol..

[46] Benjamini Y, Hochberg Y (1995). Controlling the false discovery rate: A practical and powerful approach to multiple testing. J. R. Stat. Soc. Ser. B (Methodological).

[47] R Core Team. *R: A Language and Environment for Statistical Computing*. https://www.R-project.org/ (R Foundation for Statistical Computing, 2019).

[48] White T, van der Ende J, Nichols TE (2019). Beyond Bonferroni revisited: Concerns over inflated false positive research findings in the fields of conservation genetics, biology, and medicine. Conserv. Genet..

[49] Falush D, Stephens M, Pritchard JK (2003). Inference of population structure using multilocus genotype data: Linked loci and correlated allele frequencies. Genetics.

[50] Hubisz MJ, Falush D, Stephens M, Pritchard JK (2009). Inferring weak population structure with the assistance of sample group information. Mol. Ecol. Resour..

[51] Miller, M. A., Pfeiffer, W. & Schwartz, T. Creating the CIPRES science gateway for inference of large phylogenetic trees. in *2010 Gateway Computing Environments Workshop (GCE)* 1–8 (2010).

[52] Besnier F, Glover KA (2013). ParallelStructure: A R package to distribute parallel runs of the population genetics program STRUCTURE on multi-core computers. PLoS ONE.

[53] Li Y-L, Liu J-X (2018). StructureSelector: A web-based software to select and visualize the optimal number of clusters using multiple methods. Mol. Ecol. Resour..

[54] Puechmaille SJ (2016). The program structure does not reliably recover the correct population structure when sampling is uneven: Subsampling and new estimators alleviate the problem. Mol. Ecol. Resour..

[55] Kopelman NM, Mayzel J, Jakobsson M, Rosenberg NA, Mayrose I (2015). CLUMPAK: A program for identifying clustering modes and packaging population structure inferences across K. Mol. Ecol. Resour..

[56] Anderson EC, Dunham KK (2008). The influence of family groups on inferences made with the program structure. Mol. Ecol. Resour..

[57] Dray S, Dufour A (2007). The ade4 package: Implementing the duality diagram for ecologists. J. Stat. Softw..

[58] Levene, H. Robust tests for equality of variances. in *Contributions to Probability and Statistics: Essays in Honor of Harold Hotelling* (Olkin, I., Hotelling, H. *et al*. eds.). 278–292 (Stanford University Press, 1960).

[59] Kassambara, A. *rstatix: Pipe-Friendly Framework for Basic Statistical Tests*. R package version 0.4.0. https://CRAN.R-project.org/package=rstatix (2020).

[60] Wang J (2002). An estimator for pairwise relatedness using molecular markers. Genetics.

[61] White SL, Miller WL, Dowell SA, Bartron ML, Wagner T (2018). Limited hatchery introgression into wild brook trout (*Salvelinus fontinalis*) populations despite reoccurring stocking. Evol. Appl..

[62] Lehnert SJ (2020). Multiple decades of stocking has resulted in limited hatchery introgression in wild brook trout (*Salvelinus fontinalis*) populations of Nova Scotia. Evol. Appl..

[63] Knudsen CM (2006). Comparison of life history traits between first-generation hatchery and wild upper Yakima River spring Chinook salmon. Trans. Am. Fish. Soc..

[64] Hansen MM, Mensberg K-LD (2009). Admixture analysis of stocked brown trout populations using mapped microsatellite DNA markers: Indigenous trout persist in introgressed populations. Biol. Lett..

[65] Christie MR, Ford MJ, Blouin MS (2014). On the reproductive success of early-generation hatchery fish in the wild. Evol. Appl..

[66] Fraser DJ (2019). Population correlates of rapid captive-induced maladaptation in a wild fish. Evol. Appl..

[67] Fischer JR, Kwak TJ, Flowers HJ, Cope WG, Rash JM, Besler DA (2019). Growth, condition, and trophic relations of stocked trout in southern Appalachian mountain streams. Trans. Am. Fish. Soc..

[68] Hendry AP, Day T (2005). Population structure attributable to reproductive time: Isolation by time and adaptation by time. Mol. Ecol..

[69] Gauthey Z (2017). Brown trout spawning habitat selection and its effects on egg survival. Ecol. Freshwater Fish.

[70] Dupont P-P, Bourret V, Bernatchez L (2007). Interplay between ecological, behavioural and historical factors in shaping the genetic structure of sympatric walleye populations (*Sander vitreus*). Mol. Ecol..

[71] Sandoval-Castillo J (2017). SWINGER: A user-friendly computer program to establish captive breeding groups that minimize relatedness without pedigree information. Mol. Ecol. Resour..

