# Supplementary material for: Temporal analysis shows relaxed genetic erosion following improved stocking practices in a subarctic transnational brown trout population
Source: Sci Rep. 2021 Aug 30;11:17396. doi: 10.1038/s41598-021-96681-1 (PMC8405680; doi:10.1038/s41598-021-96681-1)
Supplement: Supplementary file 1 — Supplementary Information. [file 41598_2021_96681_MOESM1_ESM.docx]

**Supplementary Information for:**

**Temporal analysis shows relaxed genetic erosion following improved stocking practices in a subarctic transnational brown trout population**

Cornelya F. C. Klütsch^1*^, Simo N. Maduna^1^, Natalia Polikarpova^2^, Kristin Forfang^1^, Benedicte Beddari^1^, Karl Øystein Gjelland^3^, Paul Eric Aspholm^4^, Per-Arne Amundsen^5^, Snorre B. Hagen^1*^

**S1. Additional Information on Molecular Methods**

After DNA extraction with the DNeasy Blood & Tissue kit (Qiagen, USA), 16 short tandem repeat (STR) markers were amplified [1-9]. Polymerase Chain Reaction (PCR) amplification was conducted in a 10.0 μl reaction volume comprising 5.0 µL 2x Multiplex PCR Master Mix (Qiagen, USA), 1.0 µL 10x primer mix, 0.05 µL BSA, 1.0 µL DNA template, and 2.95 µL RNase-free water. The conditions for PCR amplification were as follows: 10 min at 95°C denaturation, 28 cycles for 30 s 94°C, 30 s at 55°C/58°C annealing (depending on multiplex; detailed information on multiplexes is available in a table in Klütsch et al. 2019 [9]), 1 min at 72°C followed by 45 min 72°C extension. An Applied Biosystems 3730xl Genetic Analyzer (Applied Biosystems, UK) was used to separate fluorescently-labeled amplicons and GeneMapper 5.0 (Applied Biosystems, USA) was utilized to score allele sizes.

**Figure S1**. STRUCTURESELECTOR results for the entire data set based on STRUCTURE runs using the LOCPRIOR option. Left panel: STRUCTURESELECTOR results for the entire data set (i.e., all main river zones plus temporal samples), middle panel: STRUCTURESELECTOR results for zone E including temporal samples, right panel: STRUCTURESELECTOR results for zone I including temporal samples. The four different estimators introduced by [10] are listed on the right-hand side. The most likely number of genetic clusters is indicated by a red line.


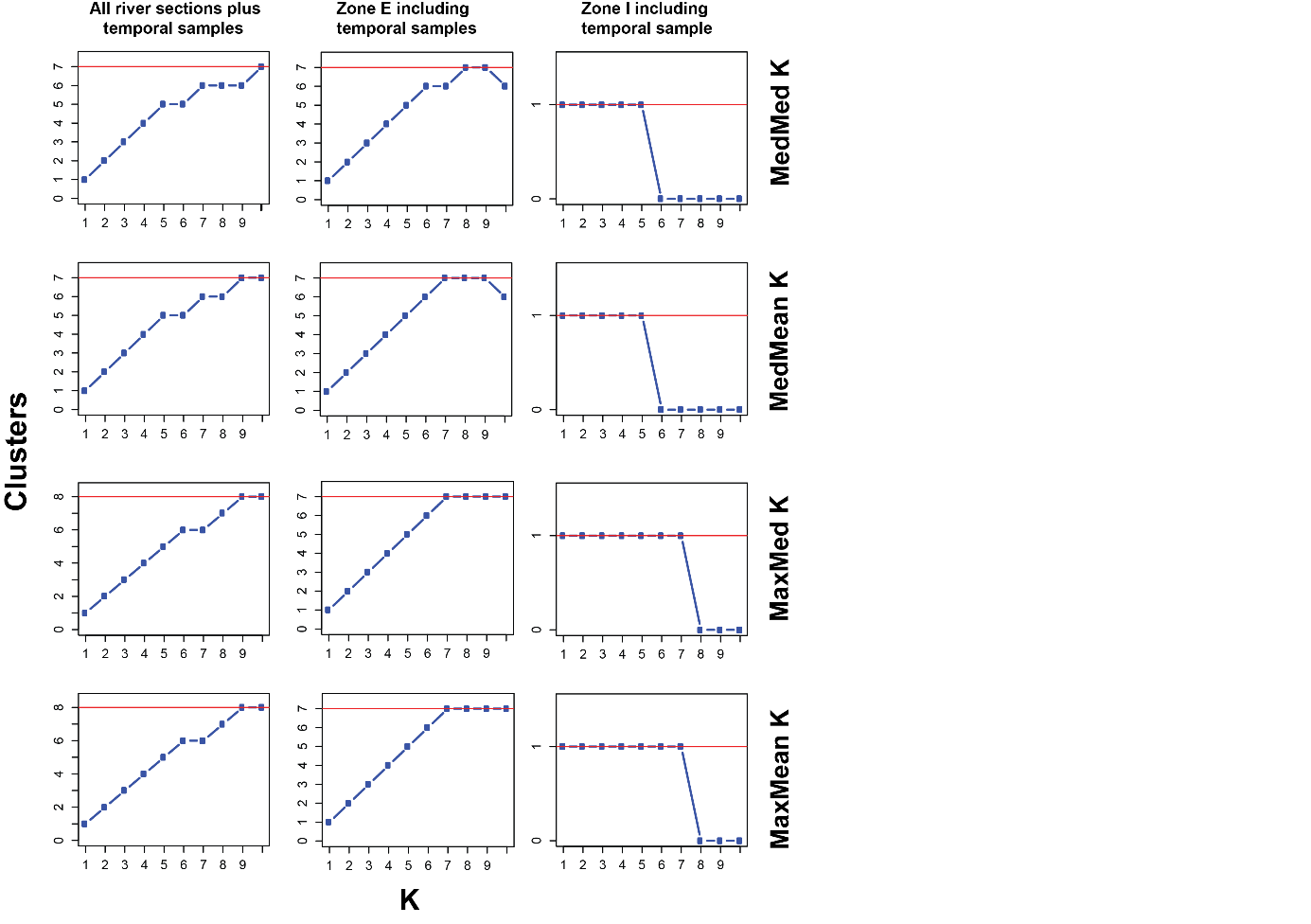


**Figure S2**. STRUCTURESELECTOR results for the reduced data set (i.e., 50 % of highly-related individuals removed) based on STRUCTURE runs. Left panel: STRUCTURESELECTOR results for the entire data set (i.e., all main river zones plus temporal samples), right panel: STRUCTURESELECTOR results for zone E including temporal samples. The four different estimators introduced by [10] are listed on the right-hand side. The most likely number of genetic clusters is indicated by a red line. Zone I had no elevated relatedness measures and therefore, no additional analysis was necessary.


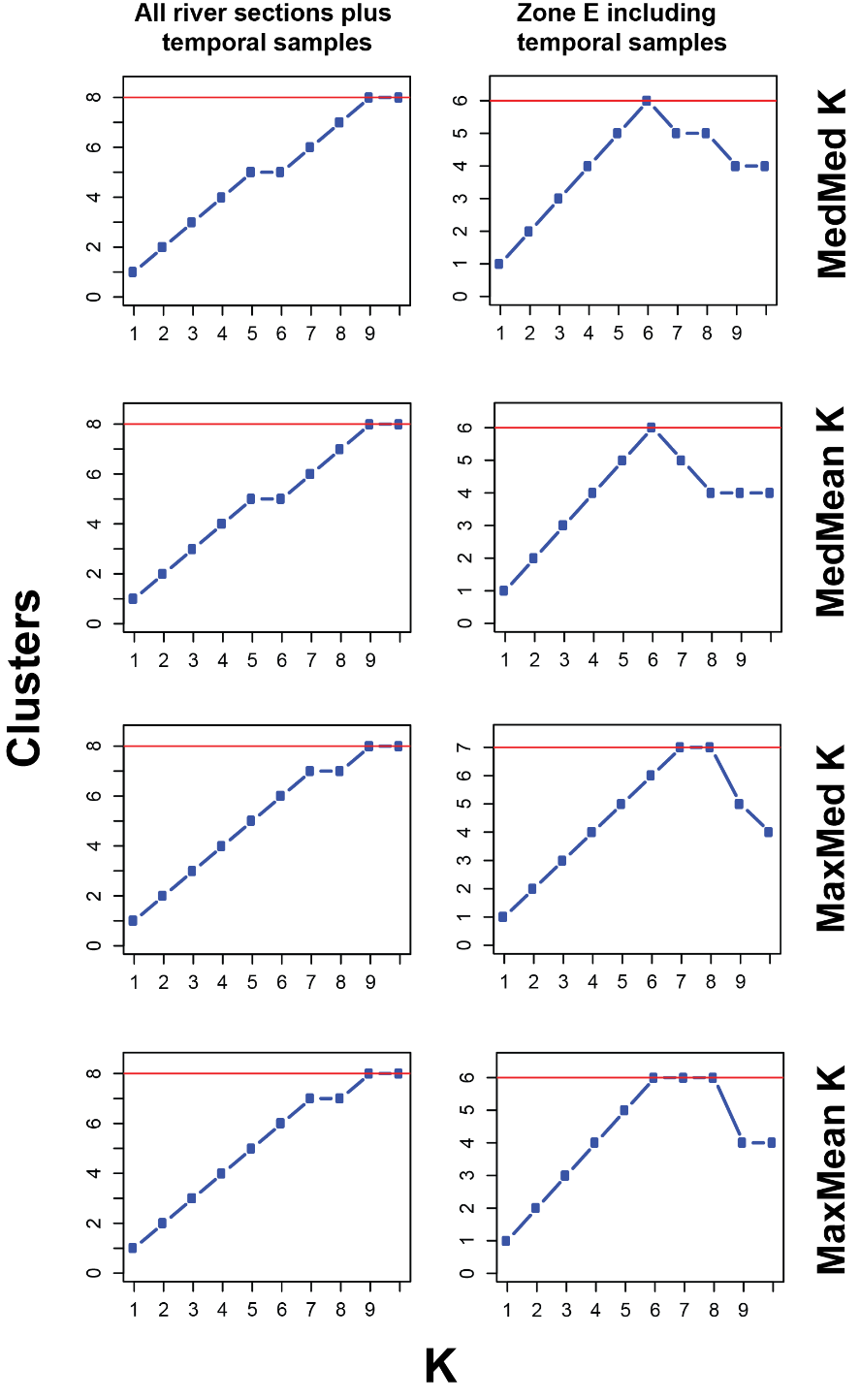


**Figure S3**. STRUCTURESELECTOR results for the reduced data set (i.e., 50 % of highly-related individuals removed) based on STRUCTURE runs using the LOCPRIOR option. Left panel: STRUCTURESELECTOR results for the entire data set (i.e., all main river zones plus temporal samples), right panel: STRUCTURESELECTOR results for zone E including temporal samples. The four different estimators introduced by [10] are listed on the right-hand side. The most likely number of genetic clusters is indicated by a red line. Zone I had no elevated relatedness measures and therefore, no additional analysis was necessary.


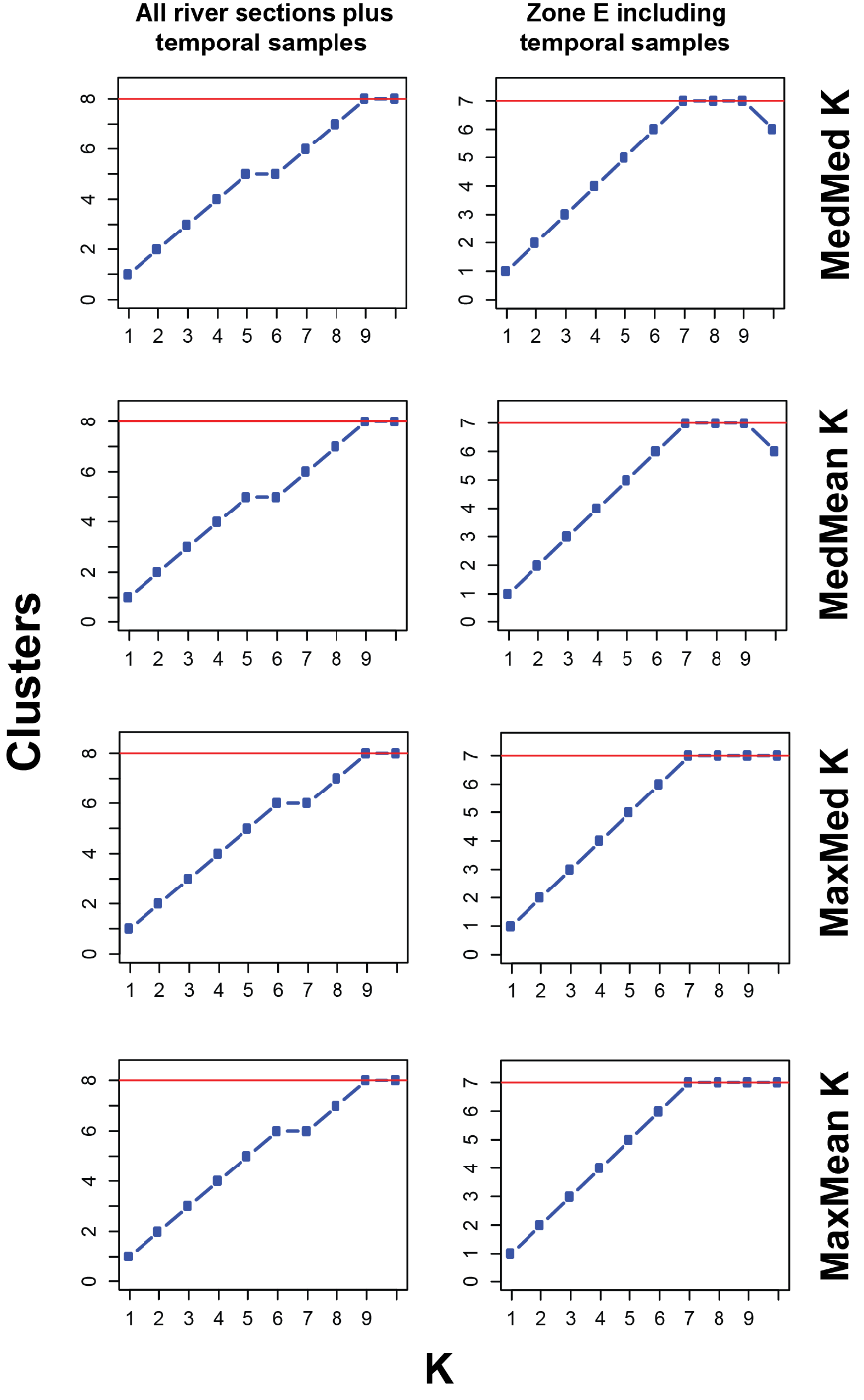


**Figure S4**. Bar plots for the different STRUCTURE runs using the LOCPRIOR option. On the left-hand side, STRUCTURE bar plots for all investigated river zones (i.e., A-J) plus temporal groups (i.e., E2008, E2007, E2001A-E2001F, and I2007) are shown. On the right-hand side, additional STRUCTURE bar plots for zones E and I are displayed that include temporal samples.


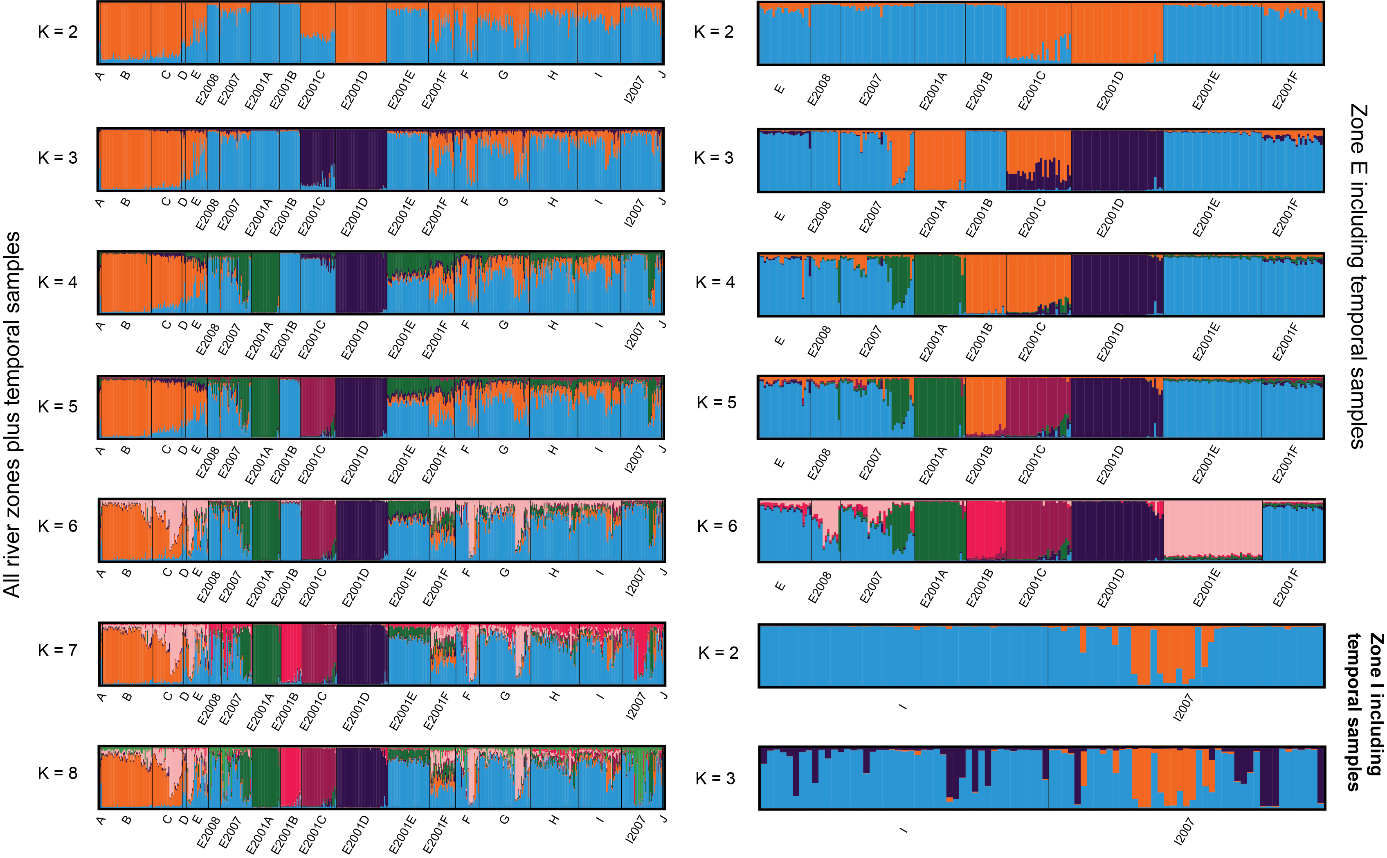


**Figure S5**. Bar plots for the STRUCTURE runs using the reduced data sets (i.e., 50 % of highly-related individuals removed). On the left-hand side, STRUCTURE bar plots for all investigated river zones (i.e., A-J) plus temporal groups (i.e., E2008, E2007, E2001A-E2001F, and I2007), are shown. On the right-hand side, additional STRUCTURE bar plots for zone E are displayed that include temporal samples. Zone I had no elevated relatedness measures and therefore, no additional analysis was necessary.


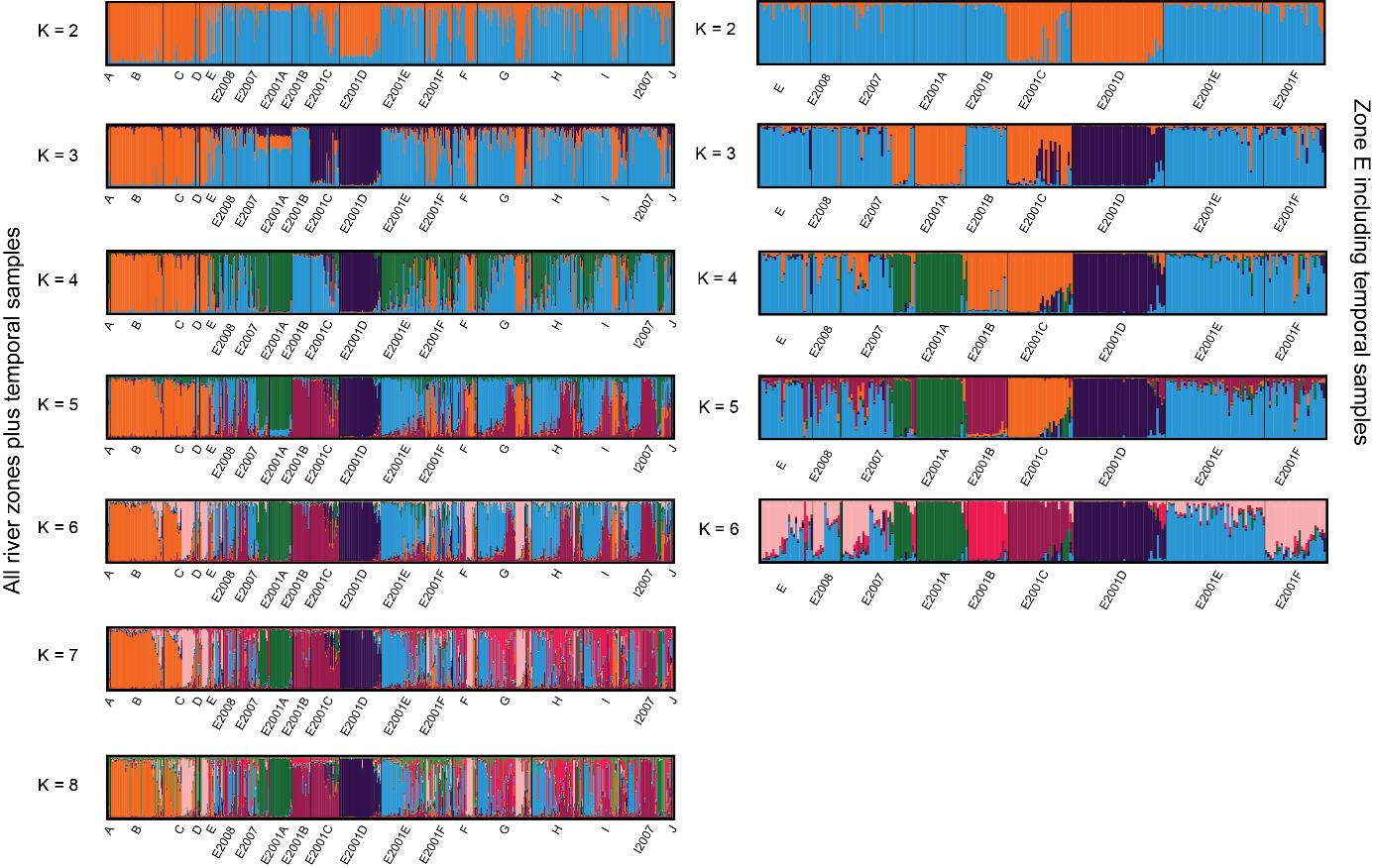


**Figure S6**. STRUCTURE bar plots for the reduced data sets (i.e., 50 % of highly-related individuals removed) using the LOCPRIOR option. On the left-hand side, STRUCTURE bar plots for all investigated river zones (i.e., A-J) plus temporal groups (i.e., E2008, E2007, E2001A-E2001F, and I2007), are shown. On the right-hand side, additional STRUCTURE bar plots for zone E are displayed that include temporal samples. Zone I had no elevated relatedness measures and therefore, no additional analysis was necessary.


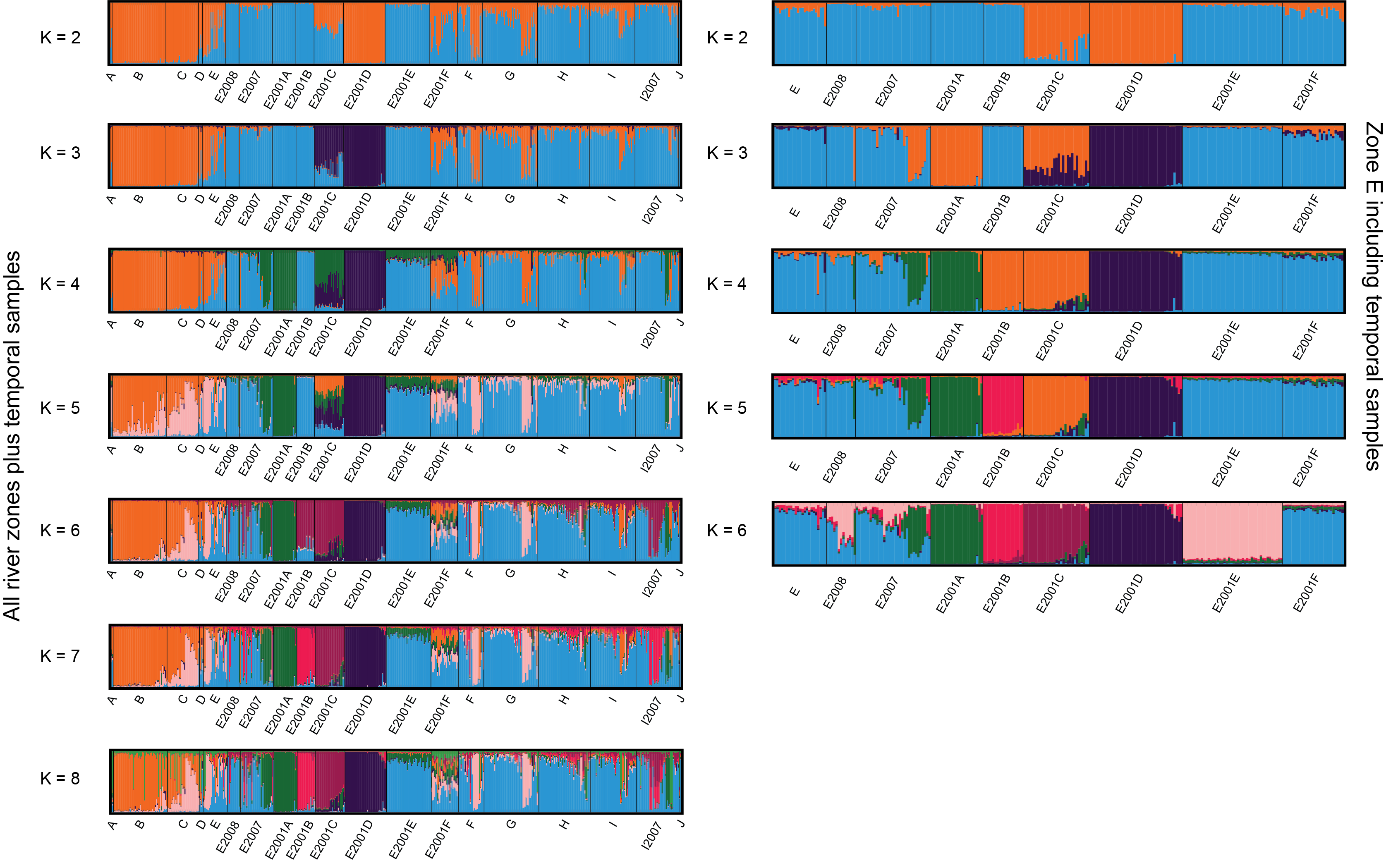


**Figure S7**. Discriminant analysis of principal components (DAPC) scatter plots based on 16 STR-markers and a reduced data set, in which highly-related individuals were removed by 50% for brown trout (*Salmo trutta*). On the left-hand side (Figures S7a-c), DAPC scatter plots for all investigated river zones (i.e., A-J) plus temporal groups (i.e., E2008, E2007, E2001A-E2001F, and I2007), are shown. On the right-hand side (Figures S7d-f), additional DAPC plots for zone E are displayed that include temporal samples. From top to bottom, three DAPC plots for principal components 1 and 2 (Figures S7a and d), 1 and 3 (Figures S7b and e), and 2 and 3 (Figures S7c and f), respectively, are shown (as per insets labelled DA eigenvalues).


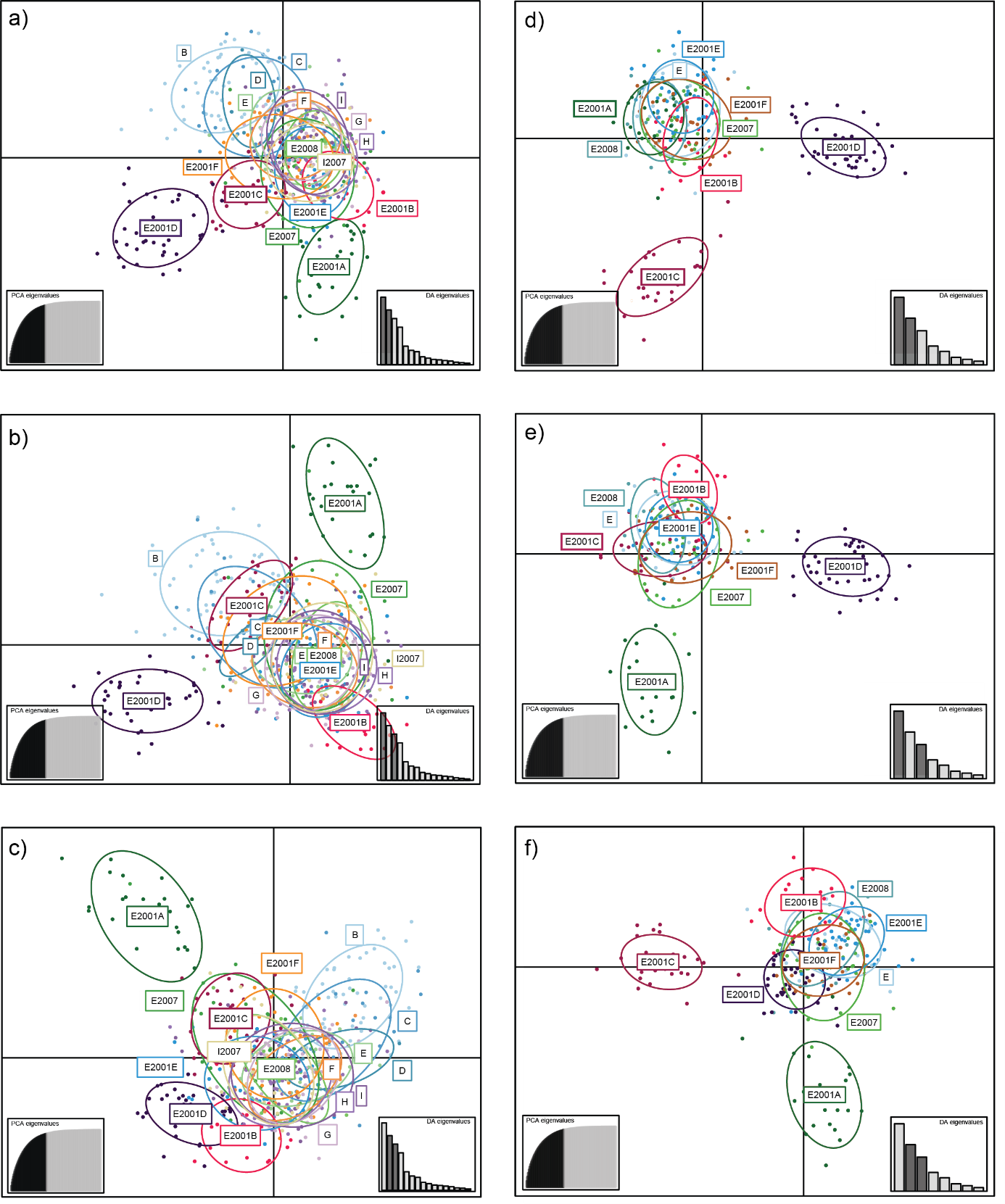


**Figure S8**. Discriminant analysis of principal components (DAPC) scatter plots for the six E2001 groups based on 16 STR-markers for brown trout (*Salmo trutta*). On the left-hand side (Figures S8a-c), DAPC scatter plots for the complete data set are shown. On the right-hand side (Figures S8d-f), the DAPC scatter plots for the reduced data set, excluding highly-related individuals by 50%, are shown. From top to bottom, three DAPC plots for principal components 1 and 2 (Fig. S8a and d), 1 and 3 (Fig. S8b and e), and 2 and 3 (Fig. S8c and f), respectively, are shown (as per insets labelled DA eigenvalues).


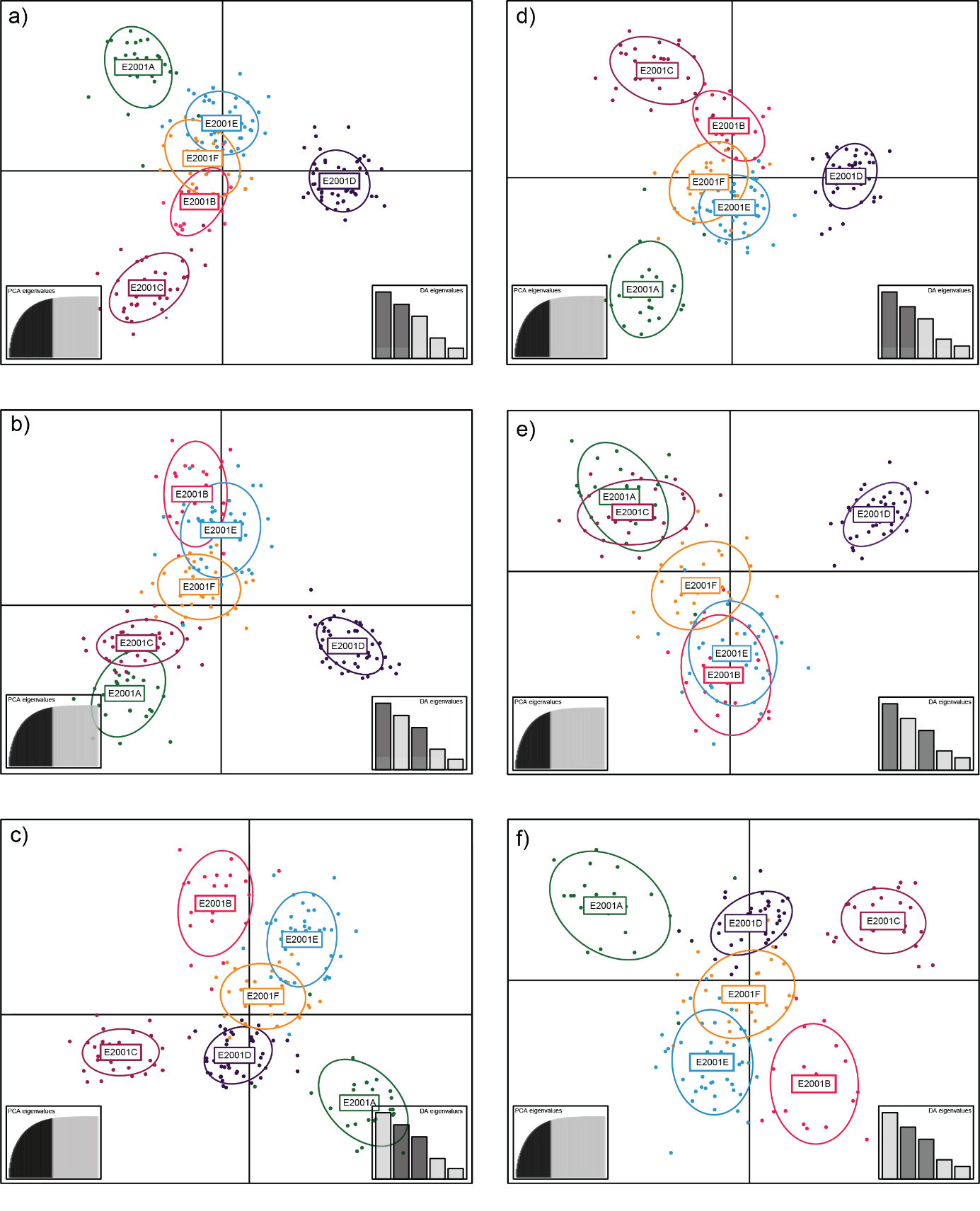


**Figure S9.** Principal Component Analysis (PCA) based on 16 STR-markers and all individuals for brown trout (*Salmo trutta*) for all investigated river zones plus temporal groups (i.e., E2008, E2007, E2001A-E2001F, and I2007). Figure S9a: PCA1 and 2; Figure S9b: PCA1 and 3; and Figure S9c: PCA2 and 3.

**
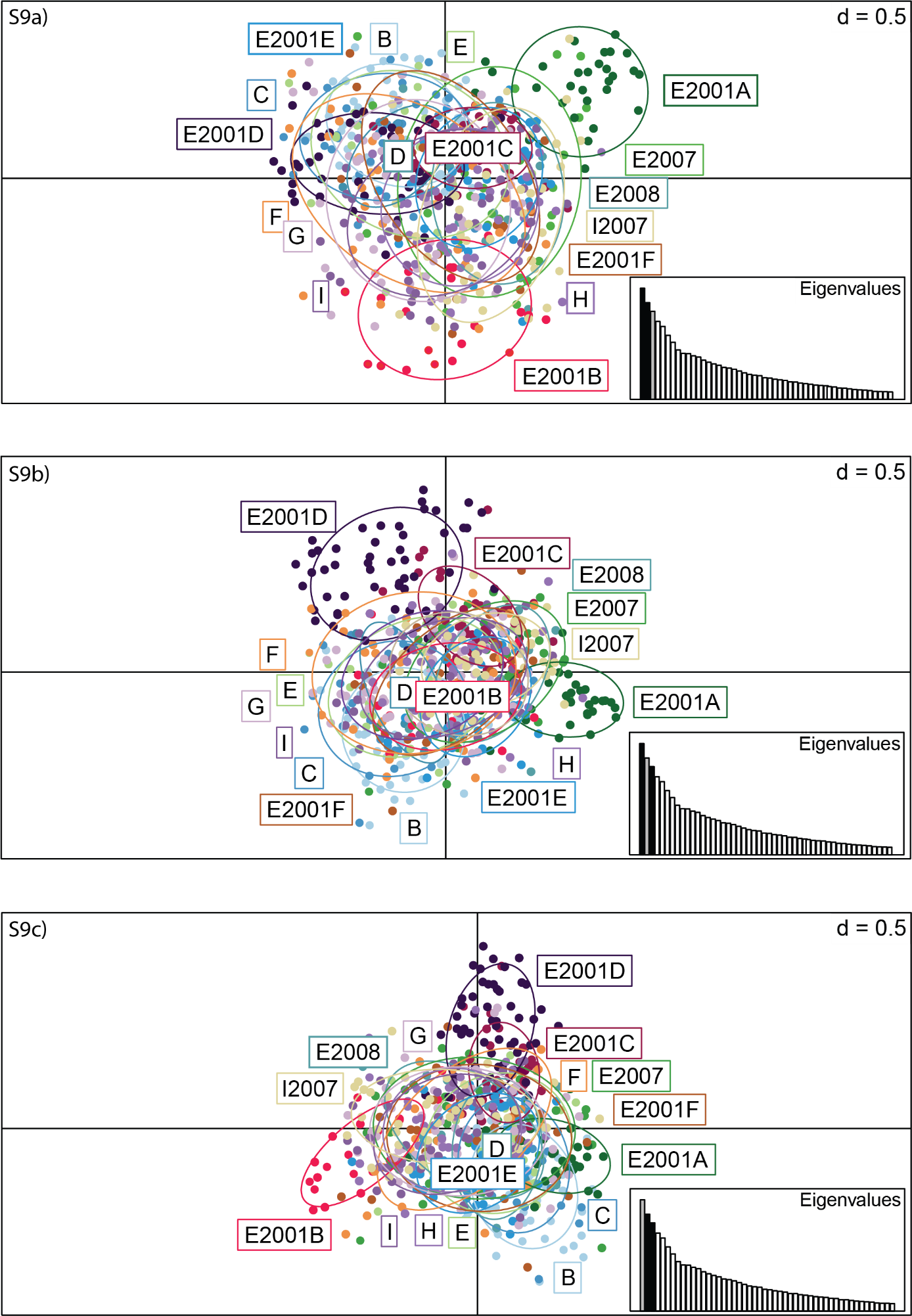
**

**Figure S10.** Principal Component Analysis (PCA) based on 16 STR-markers for brown trout (*Salmo trutta*) for all investigated river zones plus temporal groups (i.e., E2008, E2007, E2001A-E2001F, and I2007), with 50% of highly-related individuals removed. Figure S10a: PCA1 and 2; Figure S10b: PCA1 and 3; and Figure S10c: PCA2 and 3.

**
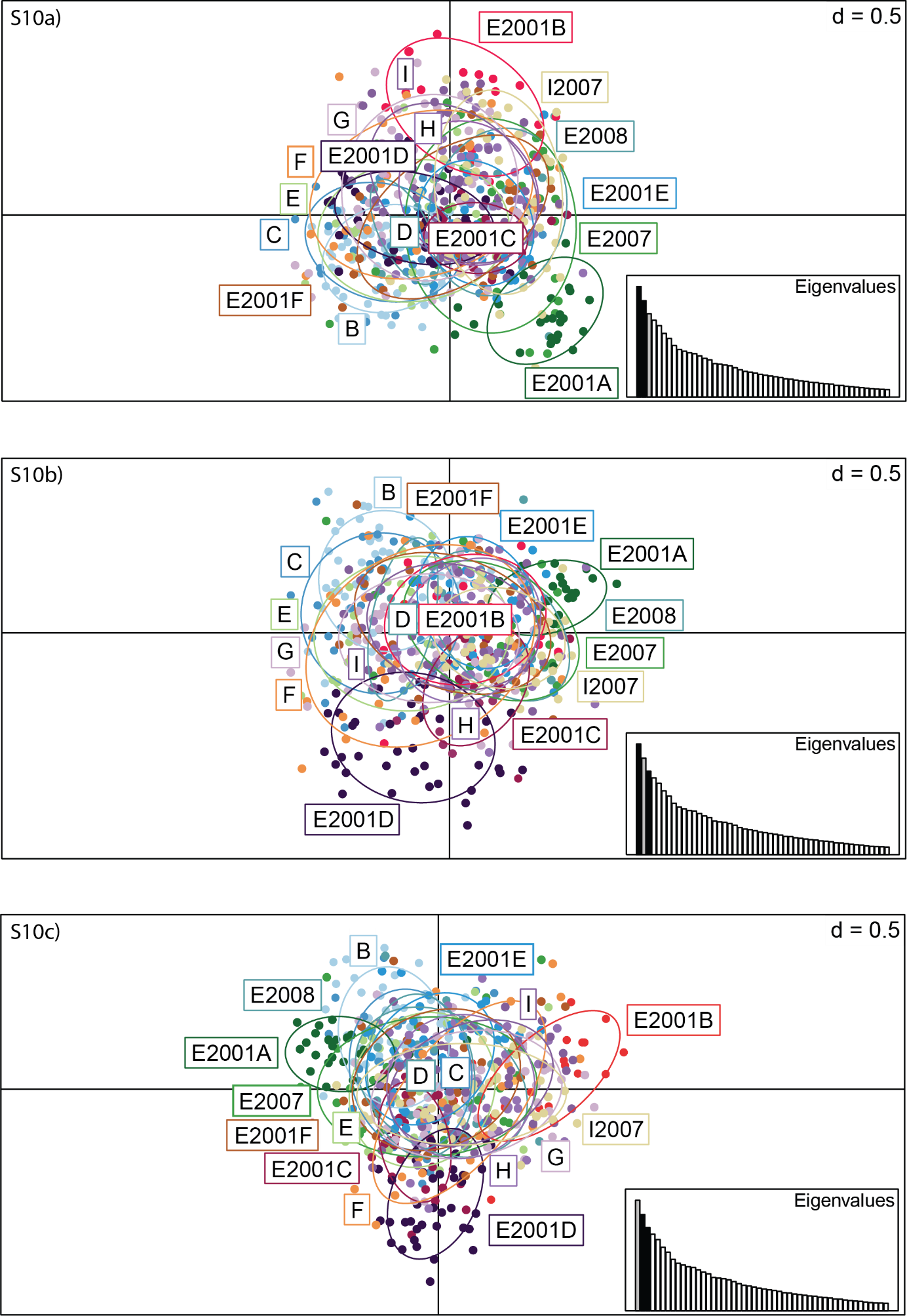
**

**Figure S11.** Principal Component Analysis (PCA) based on 16 STR-markers and all zone E individuals for brown trout (*Salmo trutta*) including temporal samples (i.e., E, E2008, E2007, and E2001A-E2001F). Figure S11a: PCA1 and 2; Figure S11b: PCA1 and 3; and Figure S11c: PCA2 and 3.

**
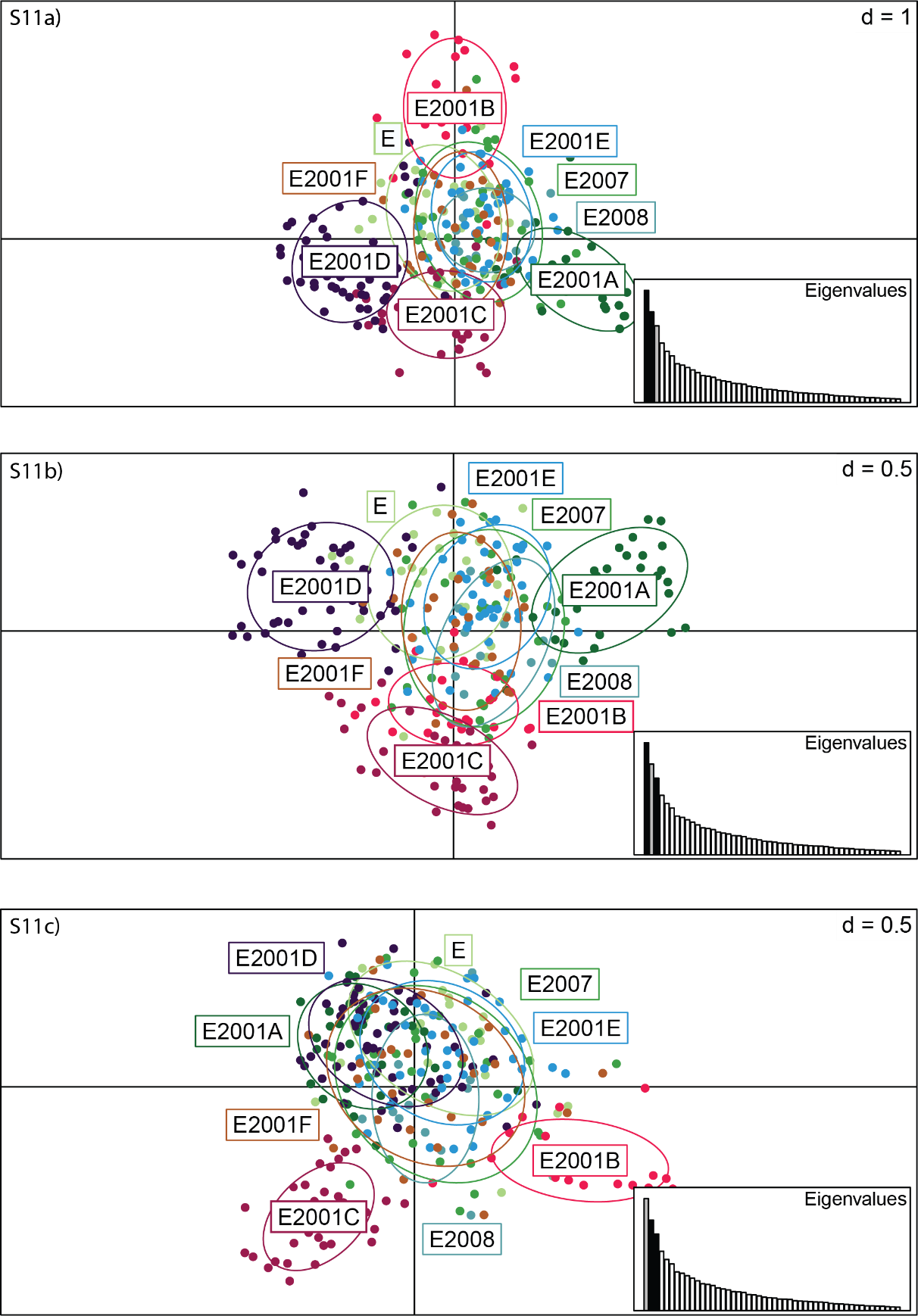
**

**Figure S12.** Principal Component Analysis (PCA) based on 16 STR-markers and E zone individuals for brown trout (*Salmo trutta*) including temporal samples E2008, E2007, and E2001A-E2001F, with 50% of highly-related individuals removed. Figure S12a: PCA1 and 2; Figure S12b: PCA1 and 3; and Figure S12c: PCA2 and 3.

**
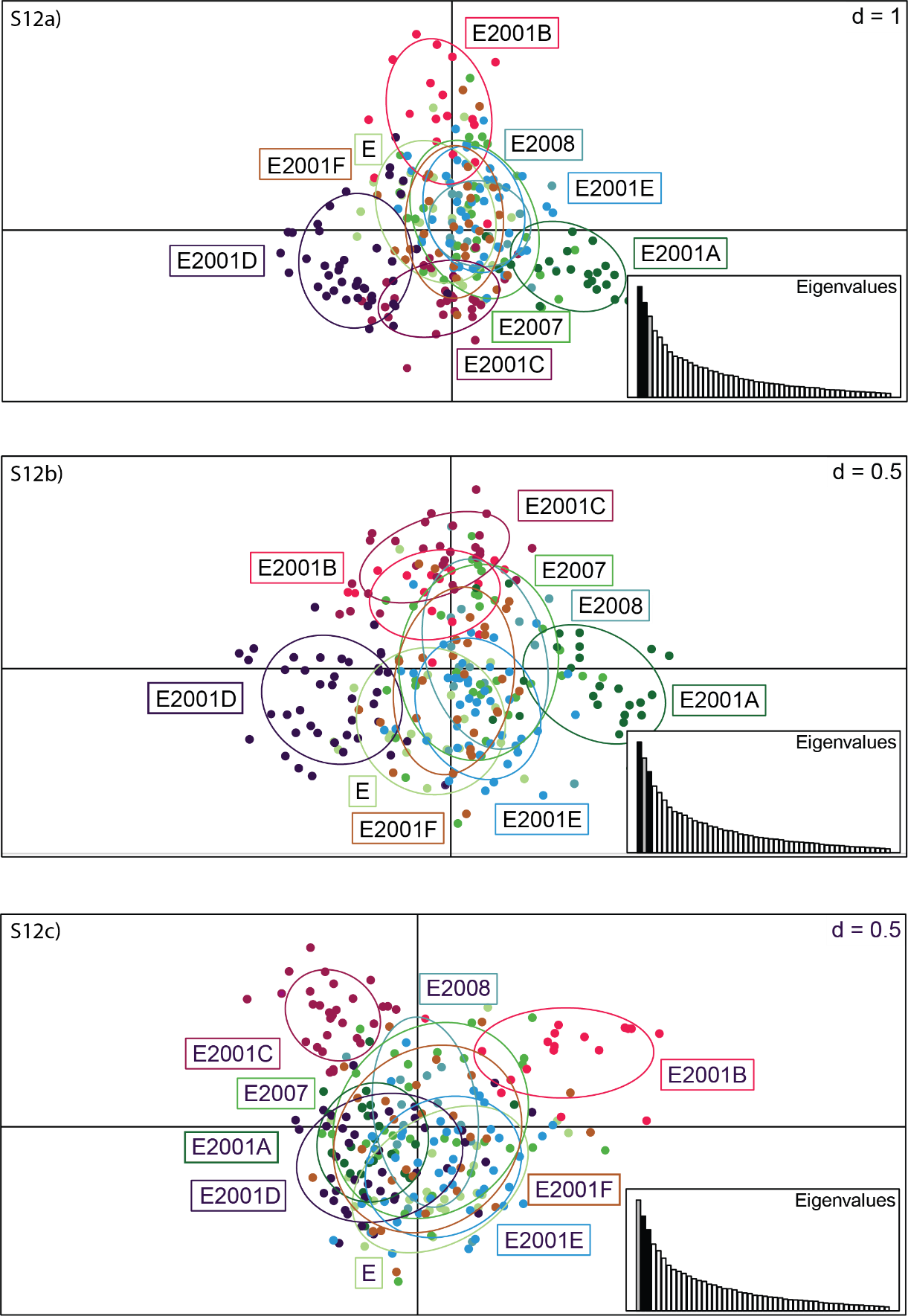
**

**Figure S13.** Principal Component Analysis (PCA) based on 16 STR-markers and all individuals for brown trout (*Salmo trutta*) in E2001A-E2001F. Figure S13a: PCA1 and 2; Figure S13b: PCA1 and 3; and Figure S13c: PCA2 and 3.

**
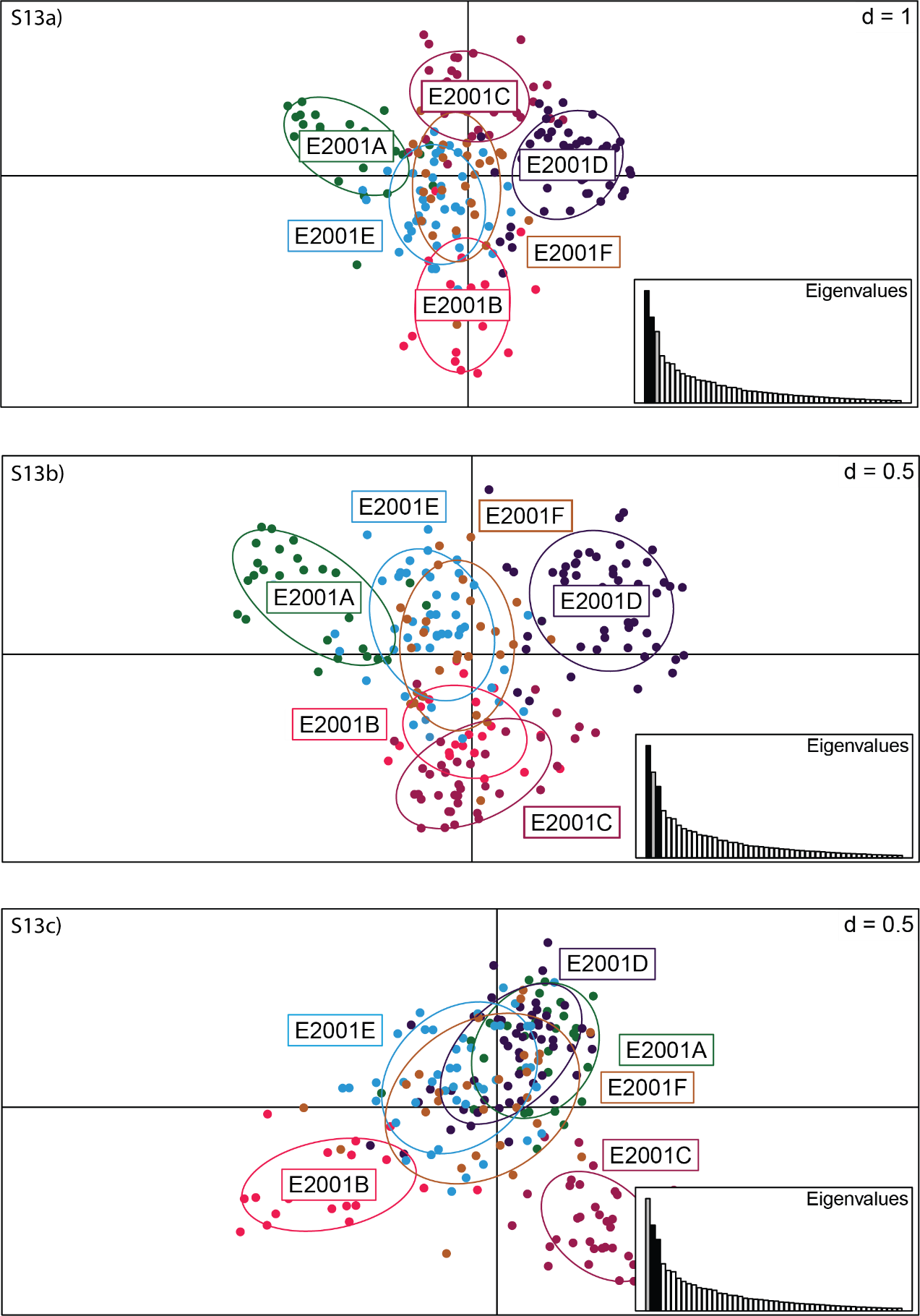
**

**Figure S14.** Principal Component Analysis (PCA) based on 16 STR-markers for brown trout (*Salmo trutta*) in E2001A-E2001F, with 50% of highly-related individuals removed. Figure S14a: PCA1 and 2; Figure S14b: PCA1 and 3; and Figure S14c: PCA2 and 3.

**
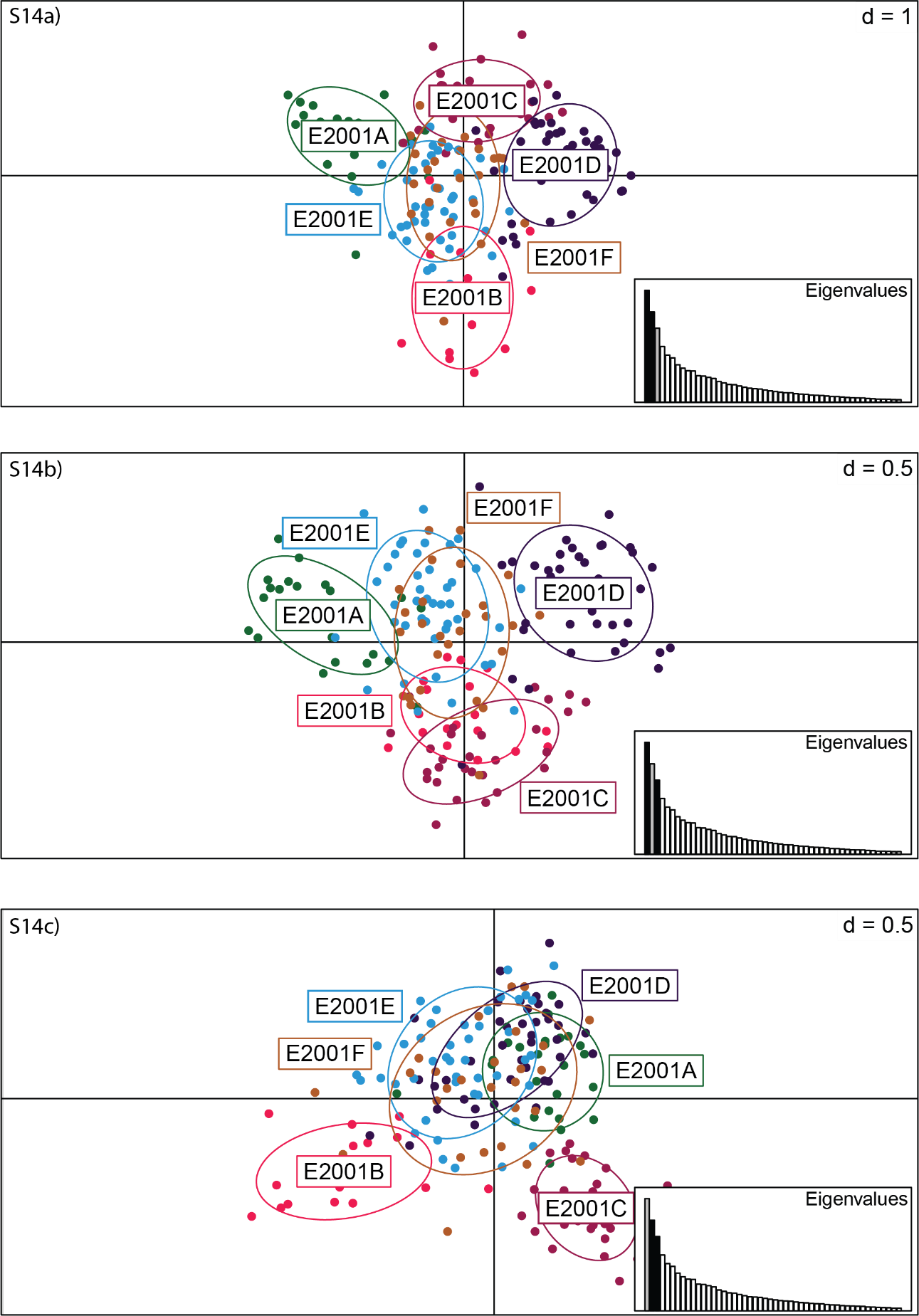
**

**Figure S15.** Pie charts showing the allele proportions for the time series in zones E and I for STR locus MST60.

**
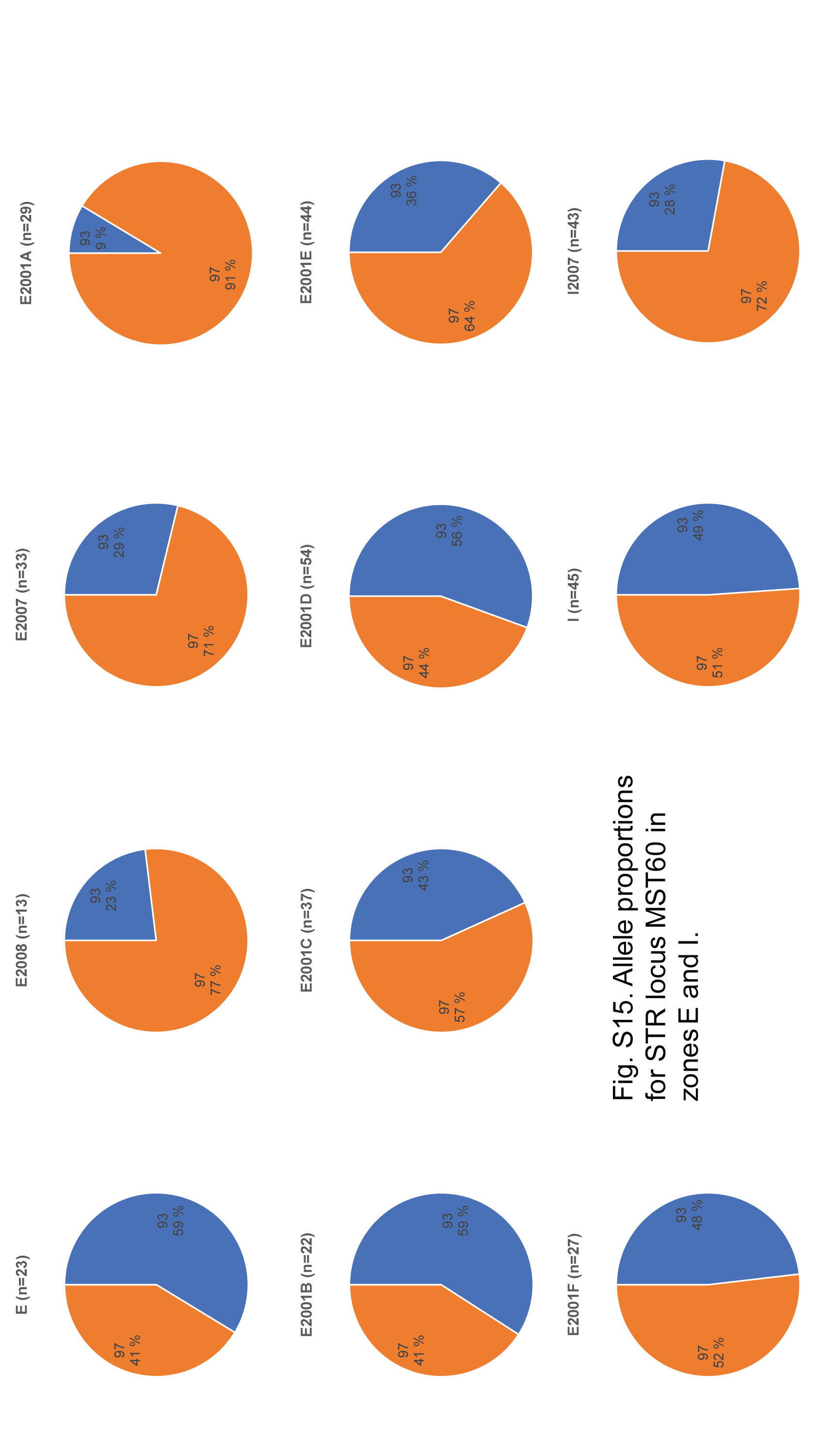
**

**Figure S16.** Pie charts showing the allele proportions for the time series in zones E and I for STR locus Strutta58.

**
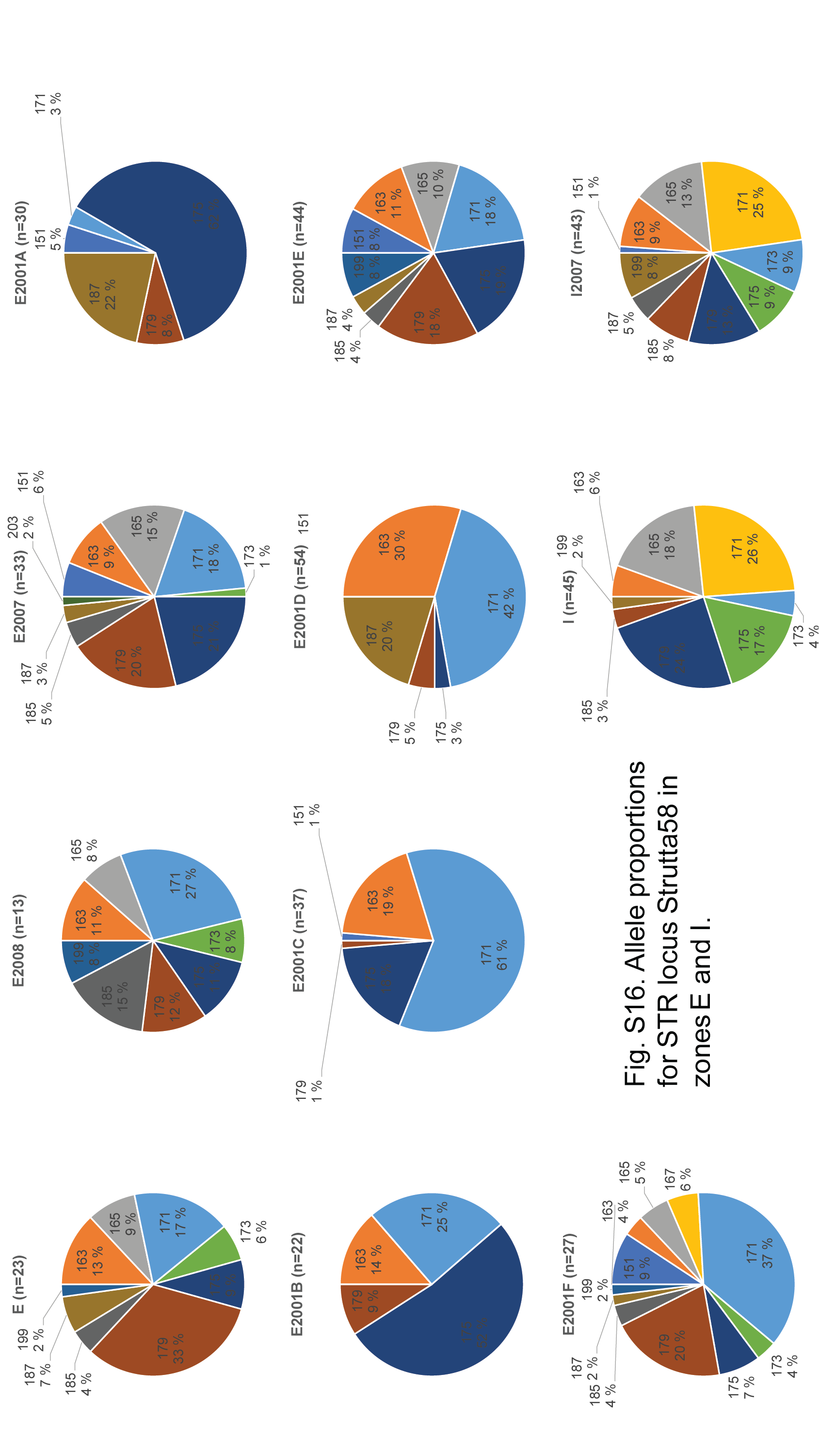
**

**Figure S17.** Pie charts showing the allele proportions for the time series in zones E and I for STR locus BHMS321.

**
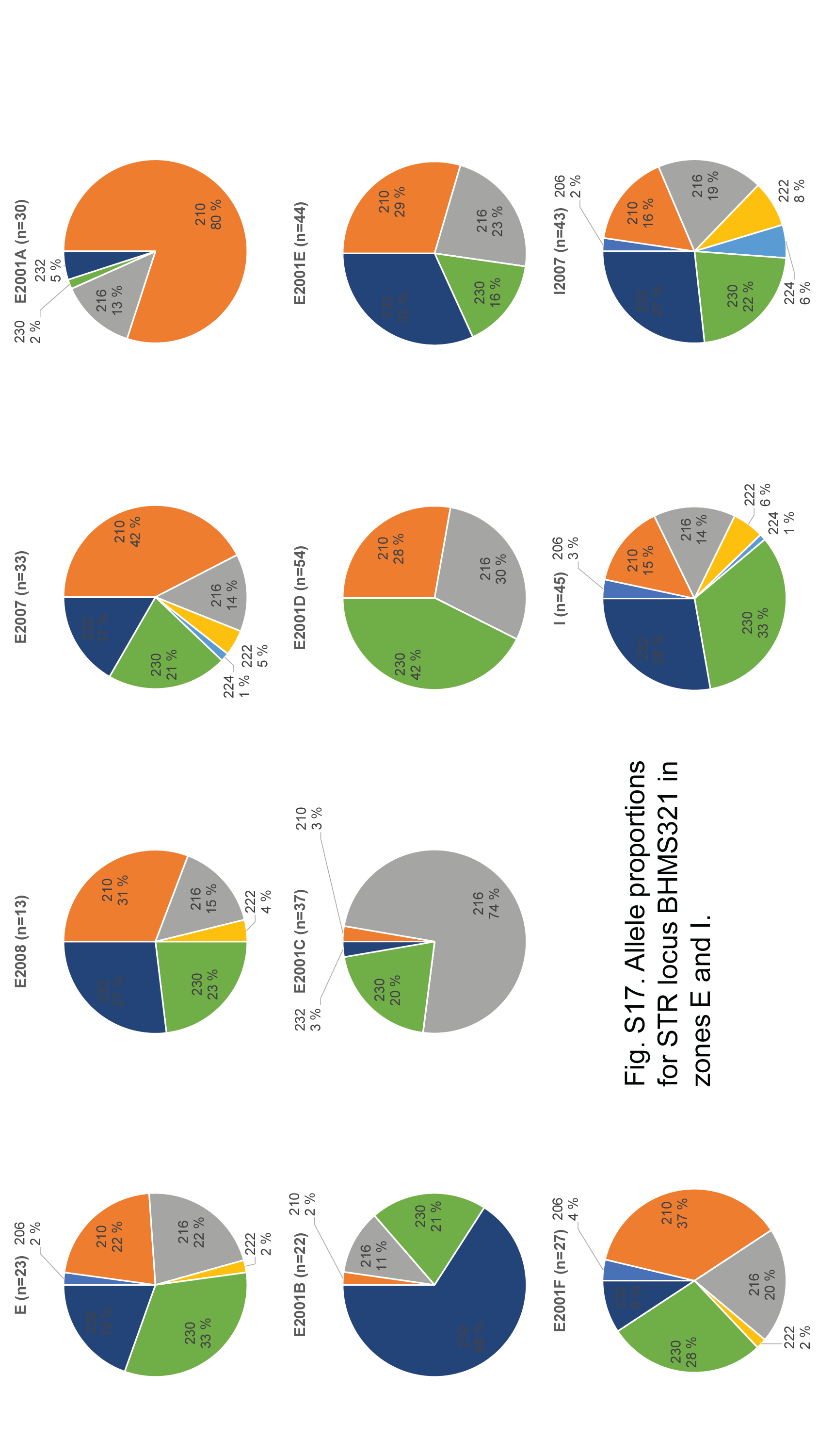
**

**Figure S18.** Pie charts showing the allele proportions for the time series in zones E and I for STR locus Ssa85.

**
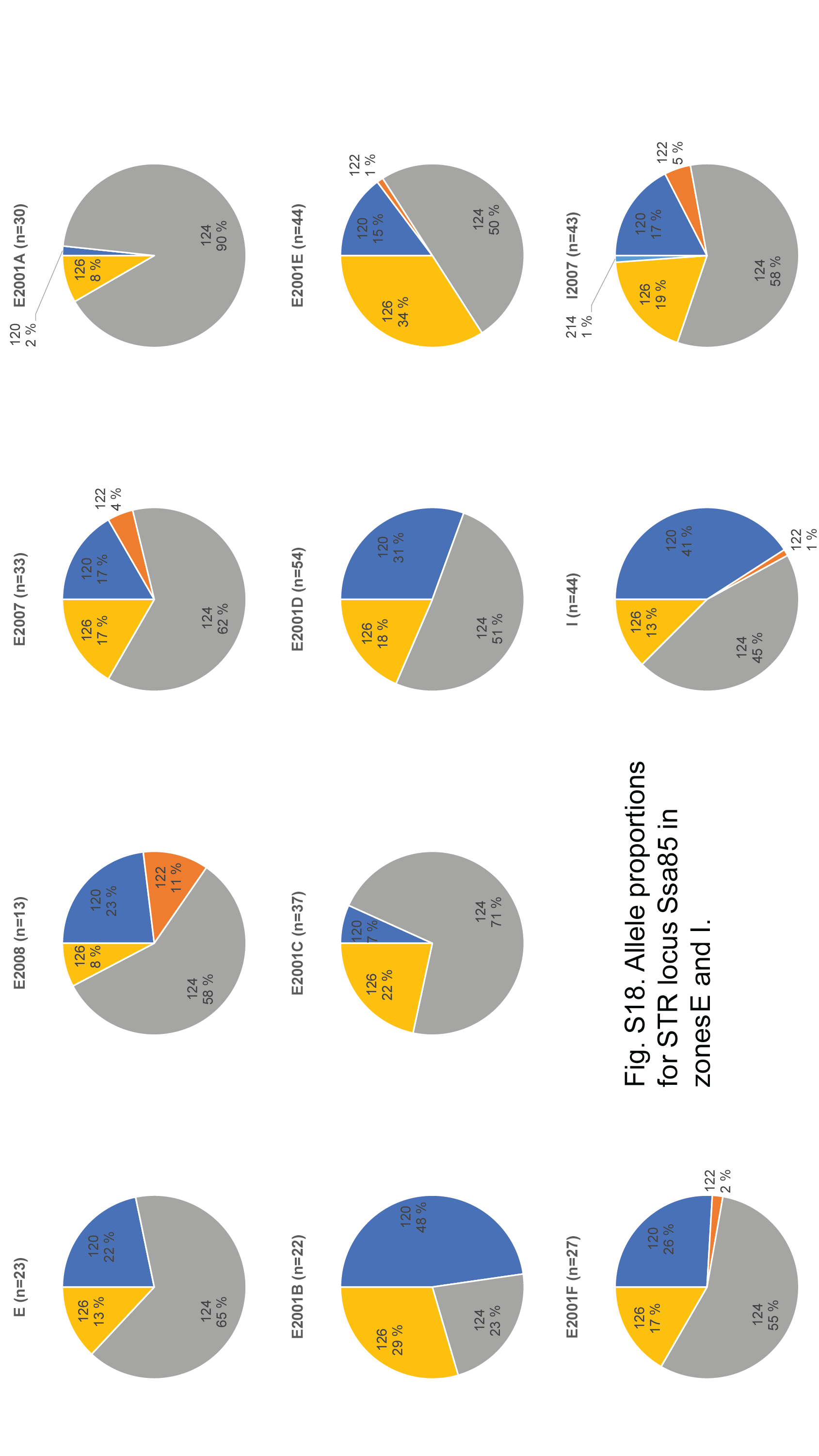
**

**Figure S19.** Pie charts showing the allele proportions for the time series in zones E and I for STR locus SsaD157.

**
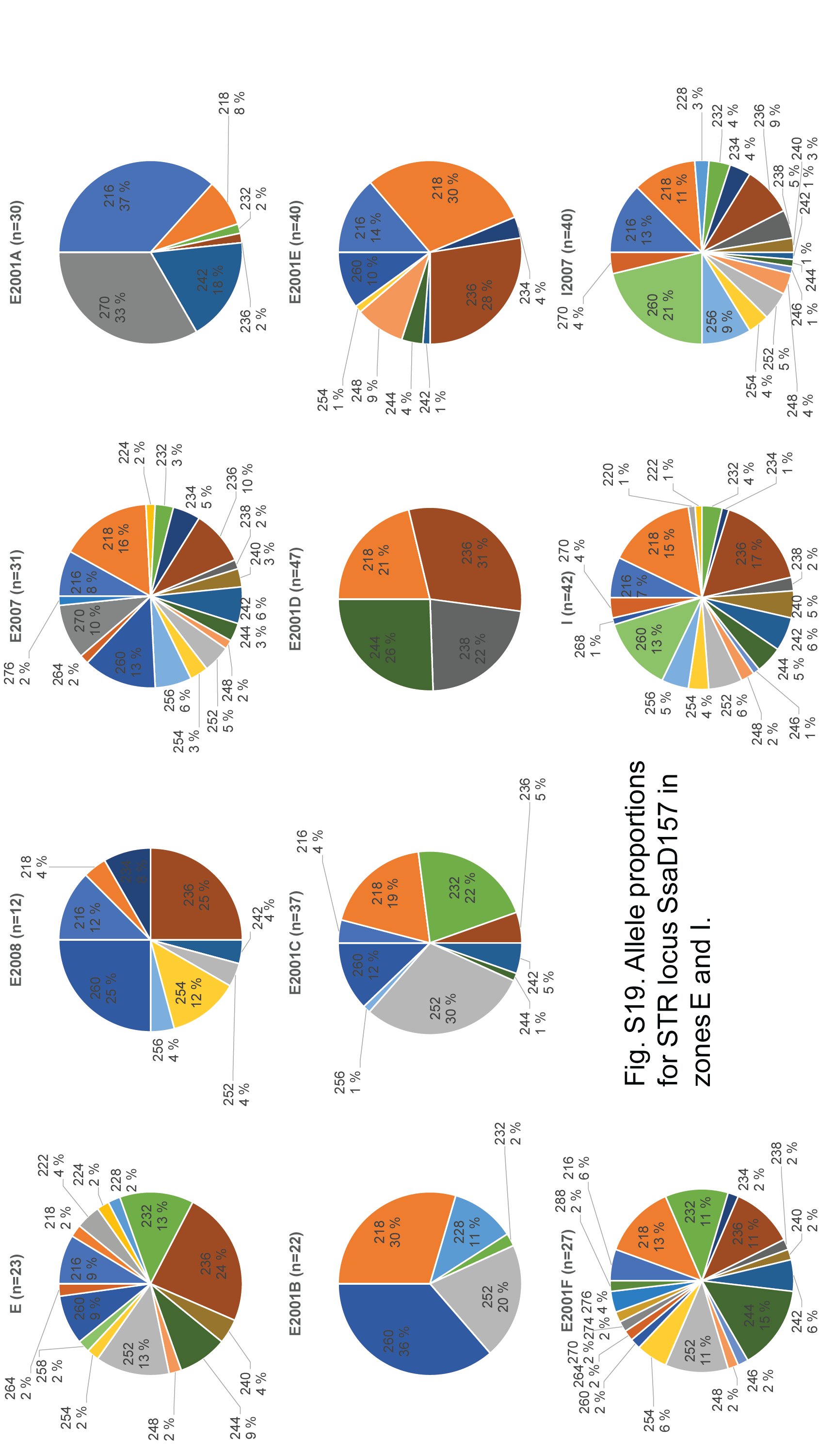
**

**Figure S20.** Pie charts showing the allele proportions for the time series in zones E and I for STR locus Ssa412UOS.

**
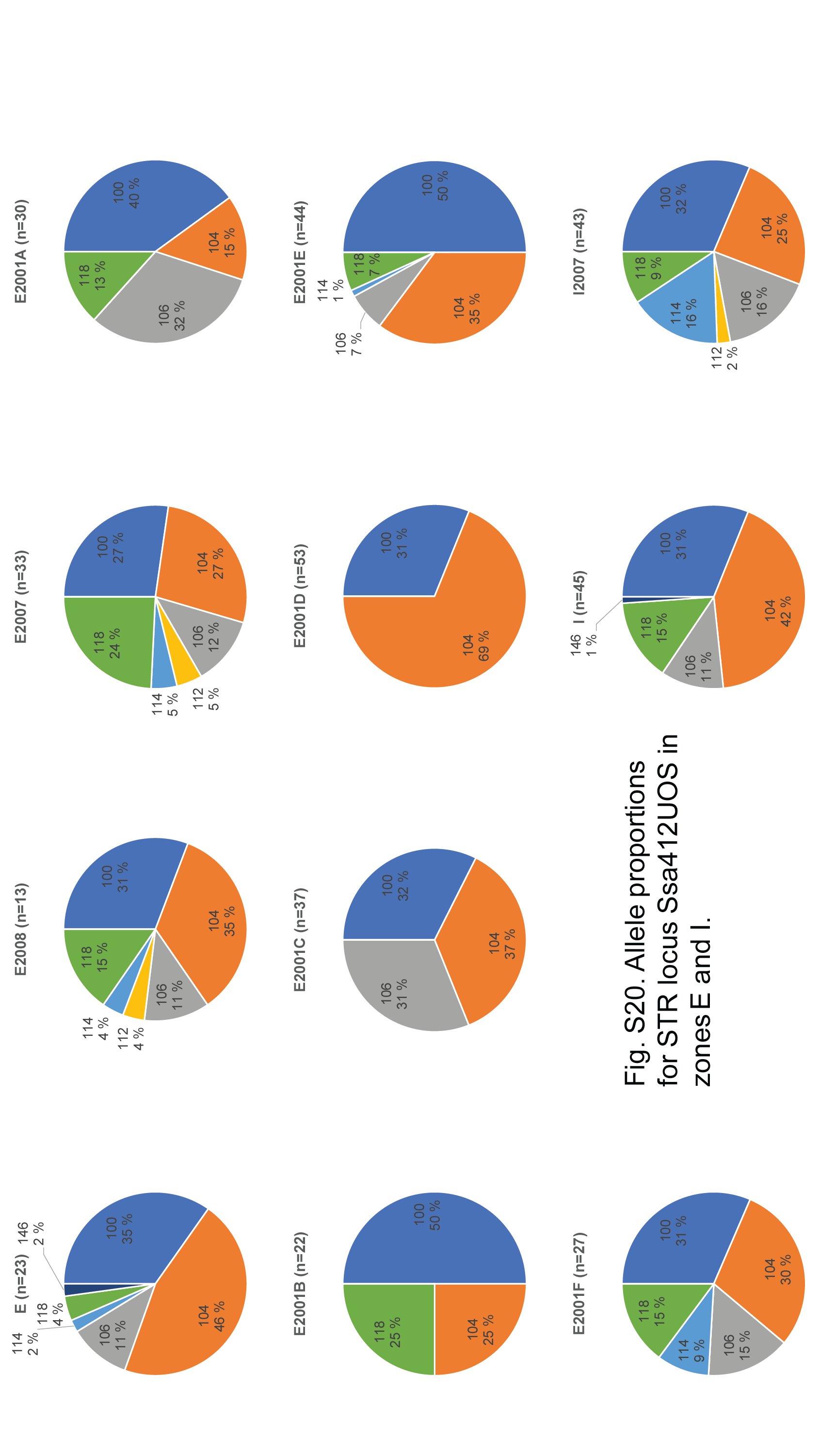
**

**Figure S21.** Pie charts showing the allele proportions for the time series in zones E and I for STR locus SSsp1605.

**
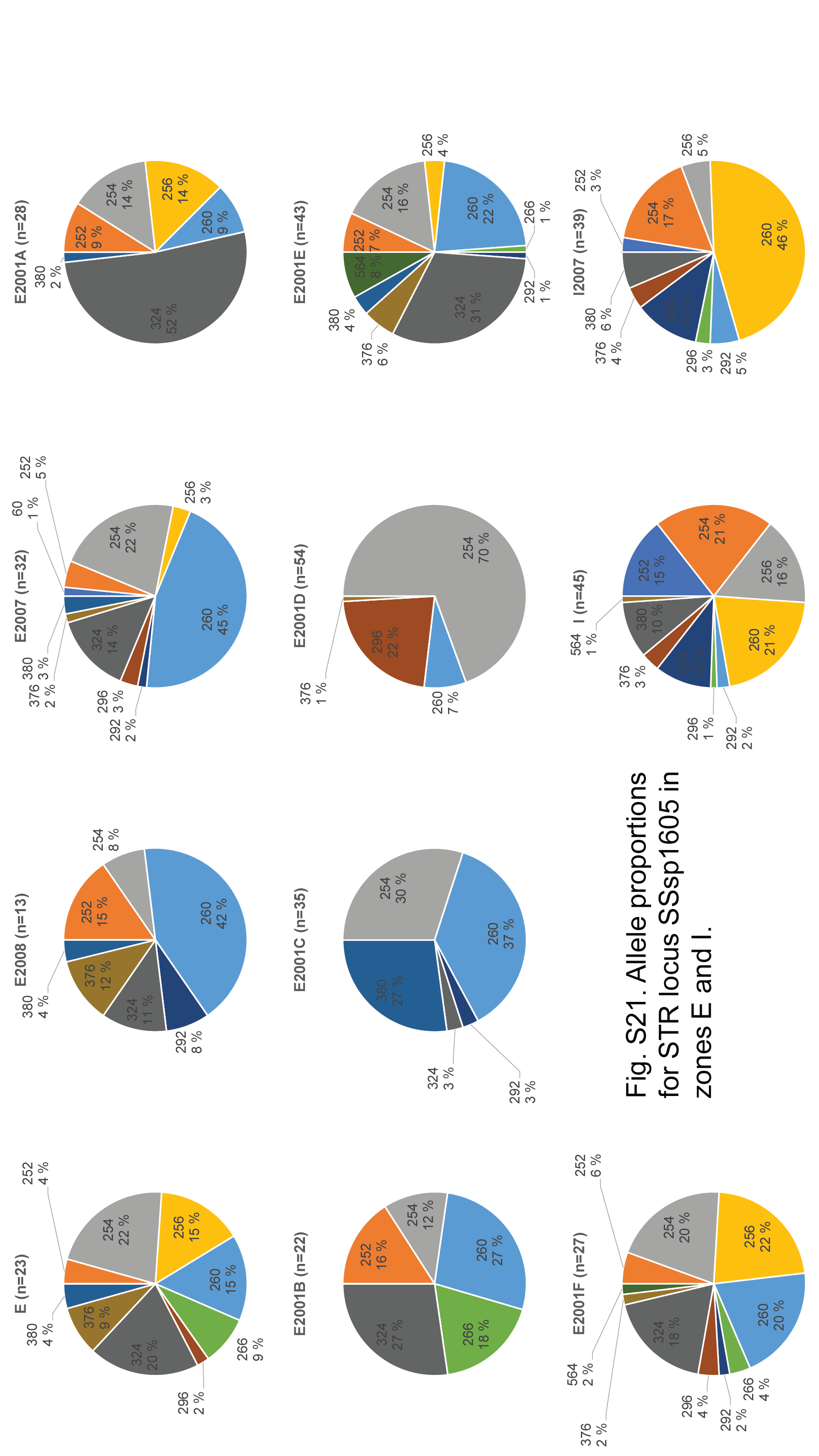
**

**Figure S22.** Pie charts showing the allele proportions for the time series in zones E and I for STR locus MST73.

**
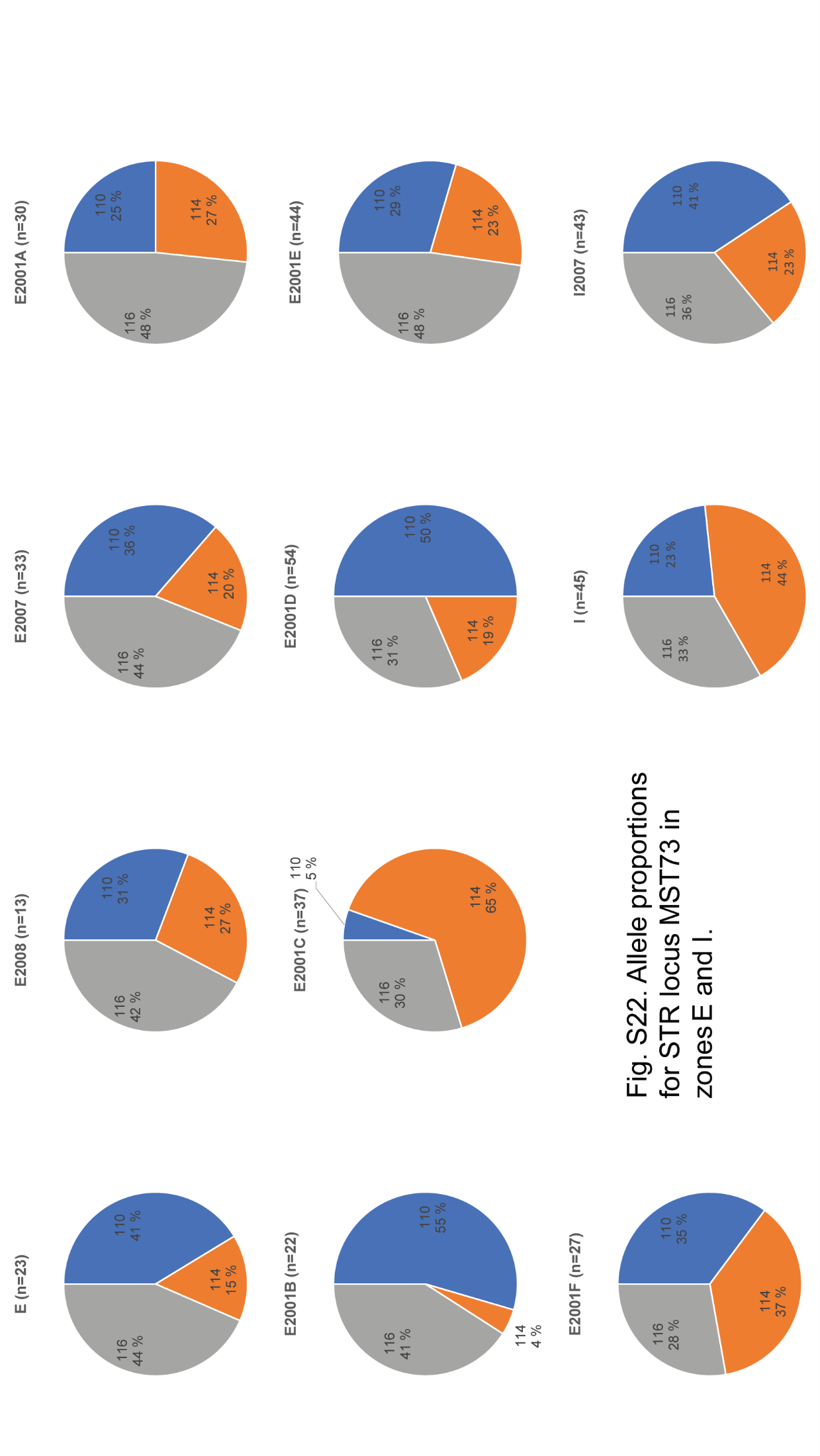
**

**Figure S23.** Pie charts showing the allele proportions for the time series in zones E and I for STR locus MST15.

**
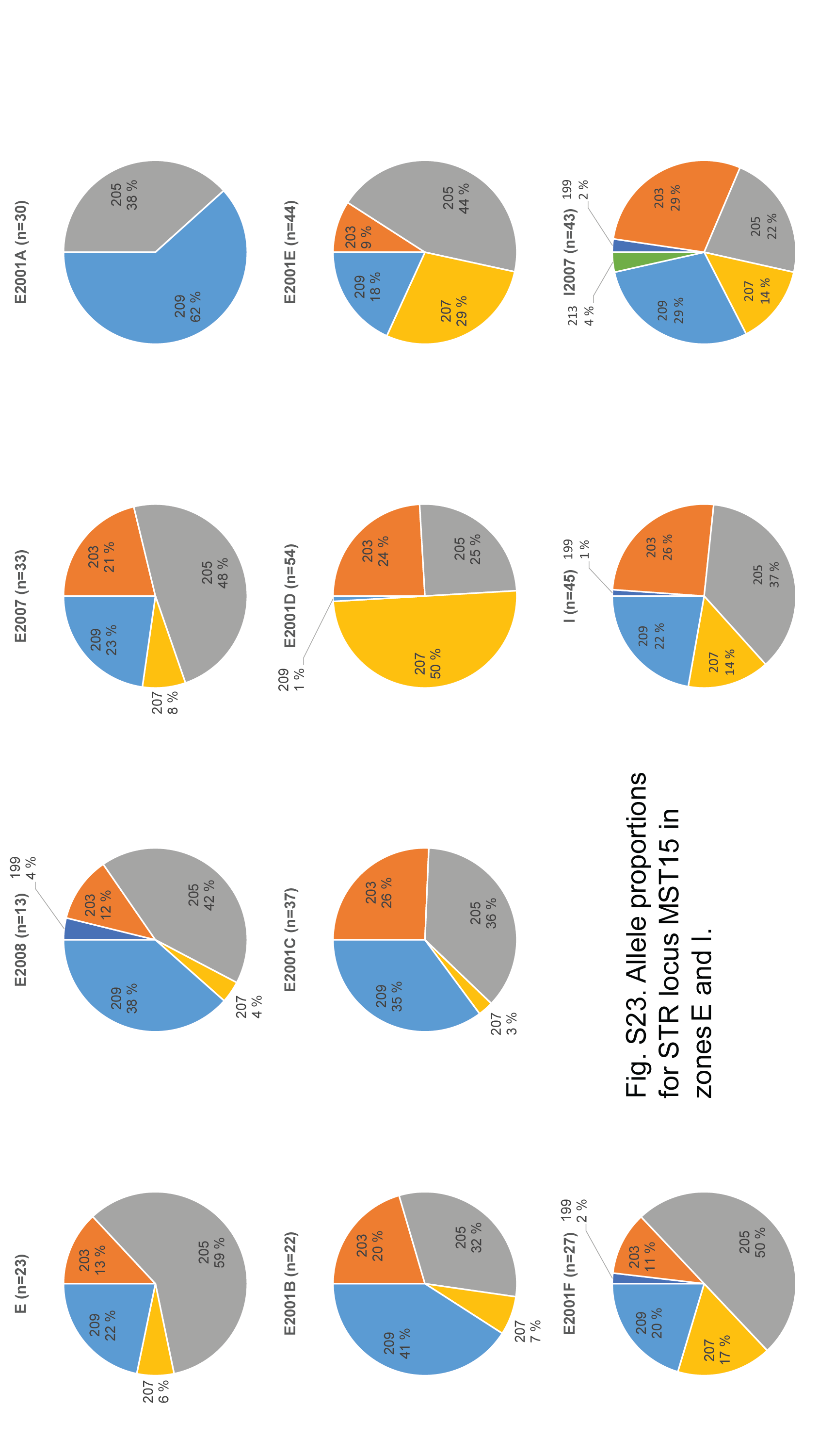
**

**Figure S24.** Pie charts showing the allele proportions for the time series in zones E and I for STR locus SsoSL85.

**
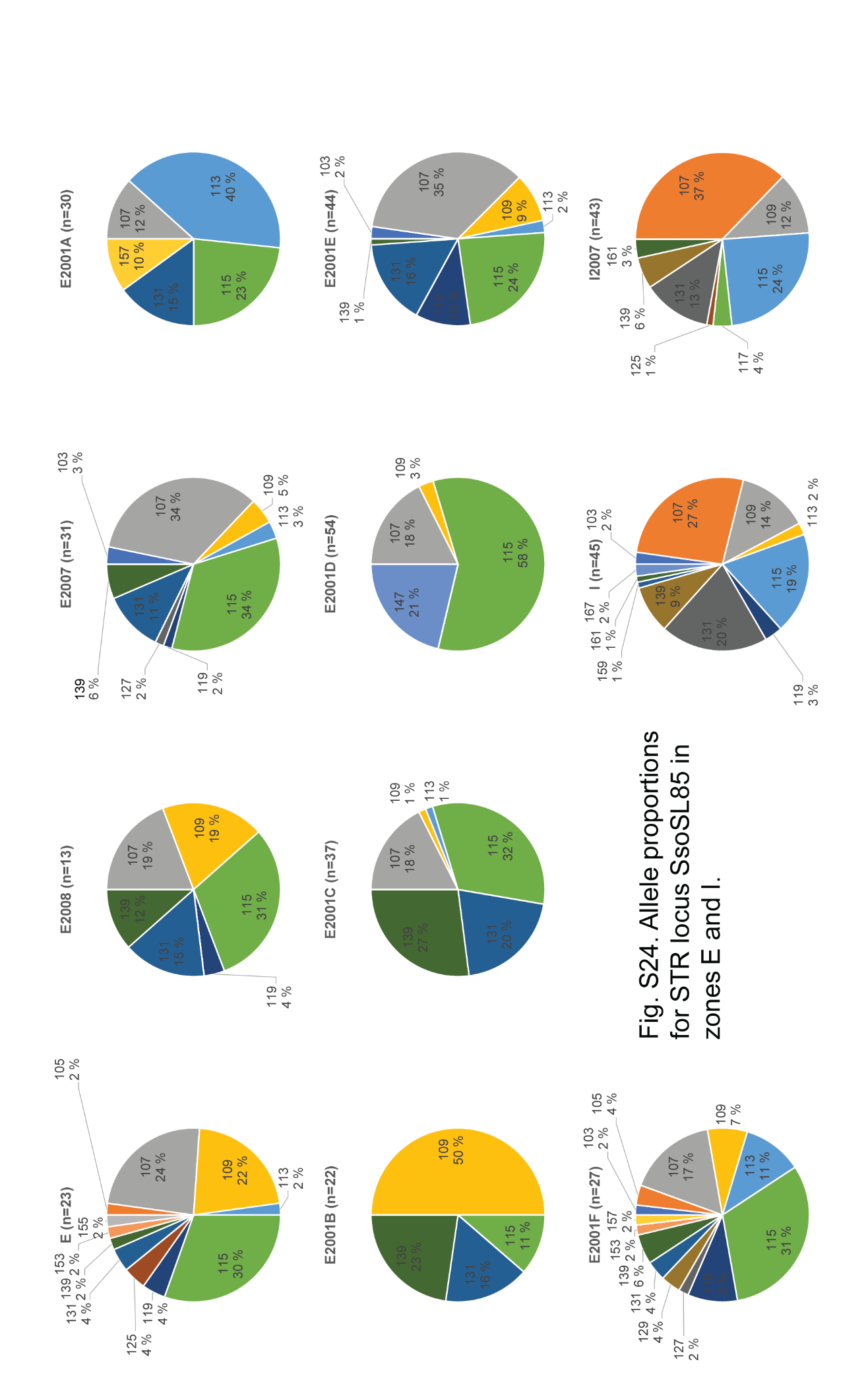
**

**Figure S25.** Pie charts showing the allele proportions for the time series in zones E and I for STR locus OMM1152.

**
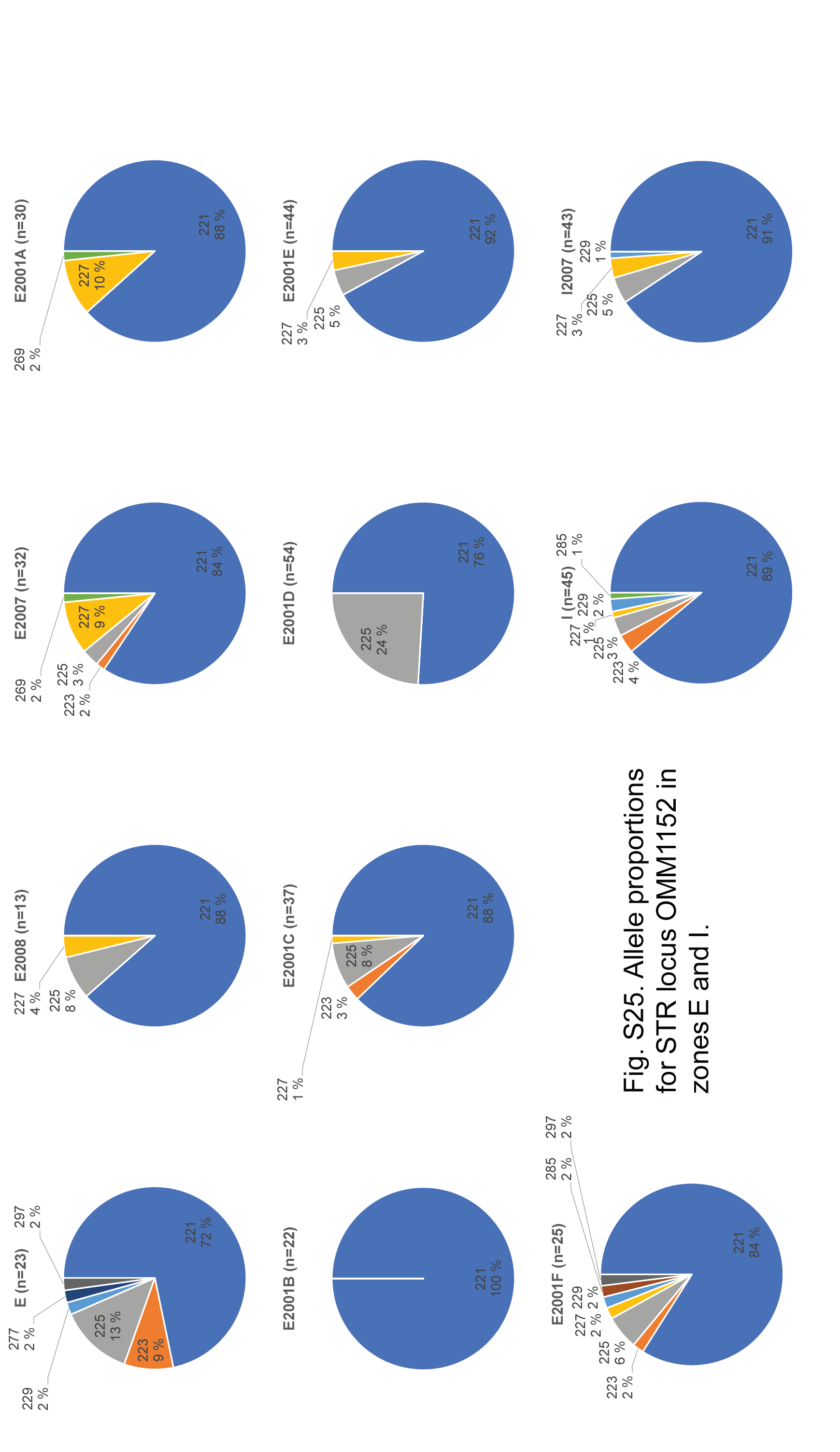
**

**Figure S26.** Pie charts showing the allele proportions for the time series in zones E and I for STR locus SSsp2216.

**
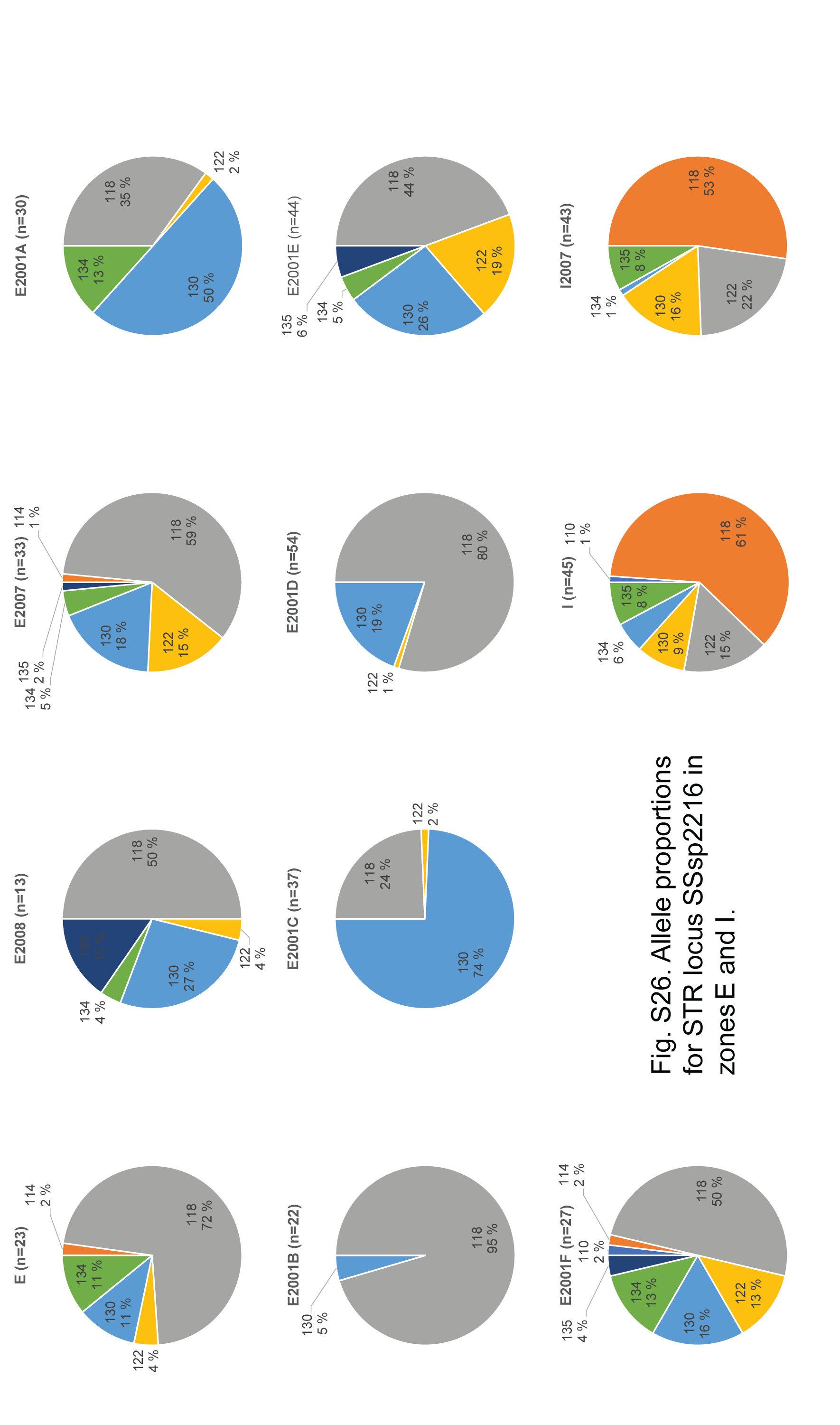
**

**Figure S27.** Pie charts showing the allele proportions for the time series in zones E and I for STR locus Ssa197.

**
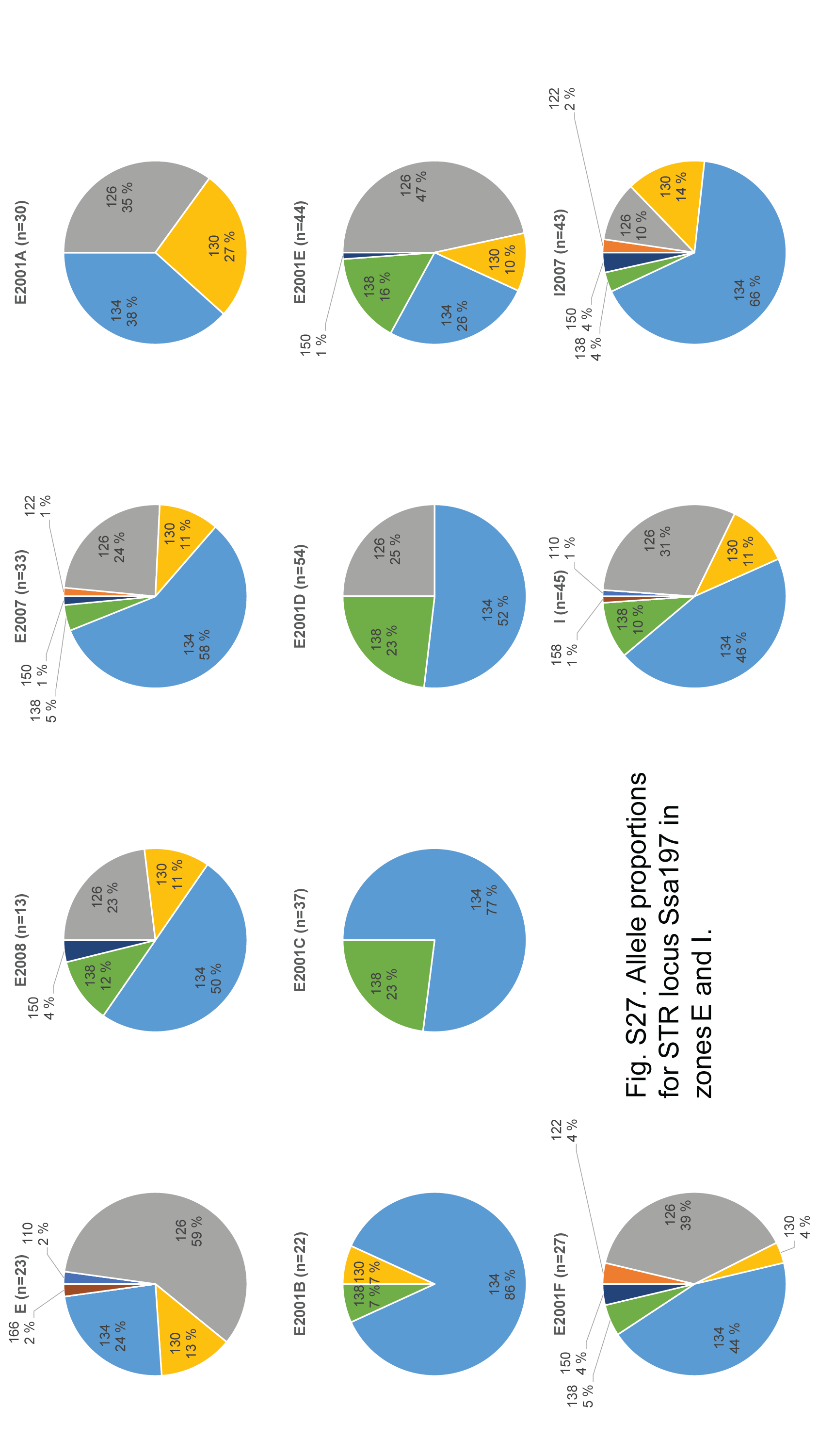
**

**Figure S28.** Pie charts showing the allele proportions for the time series in zones E and I for STR locus One102.

**
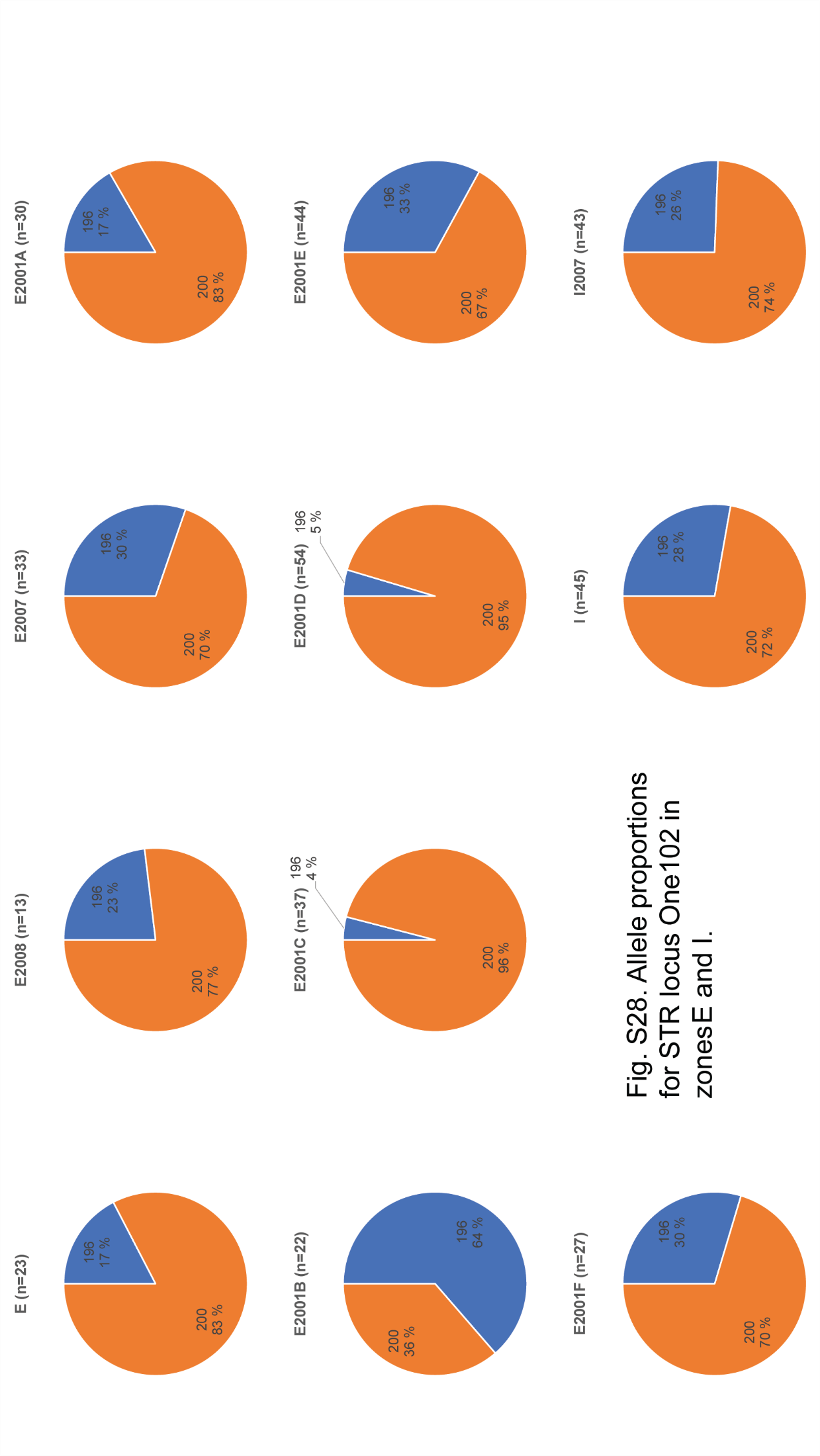
**

**Figure S29.** Pie charts showing the allele proportions for the time series in zones E and I for STR locus SsoSL438.

**
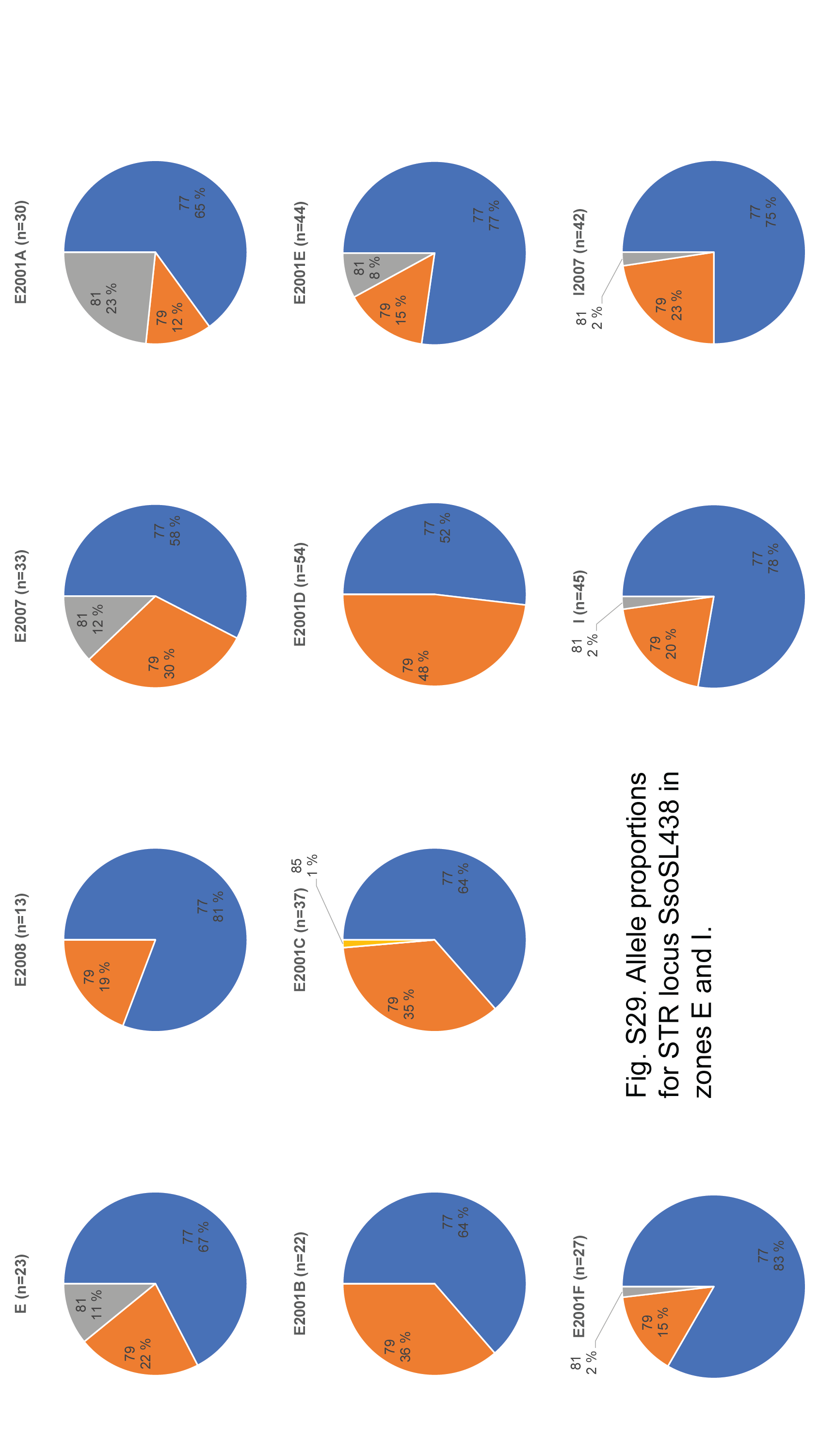
**

**Figure S30.** Pie charts showing the allele proportions for the time series in zones E and I for STR locus MST543.

**
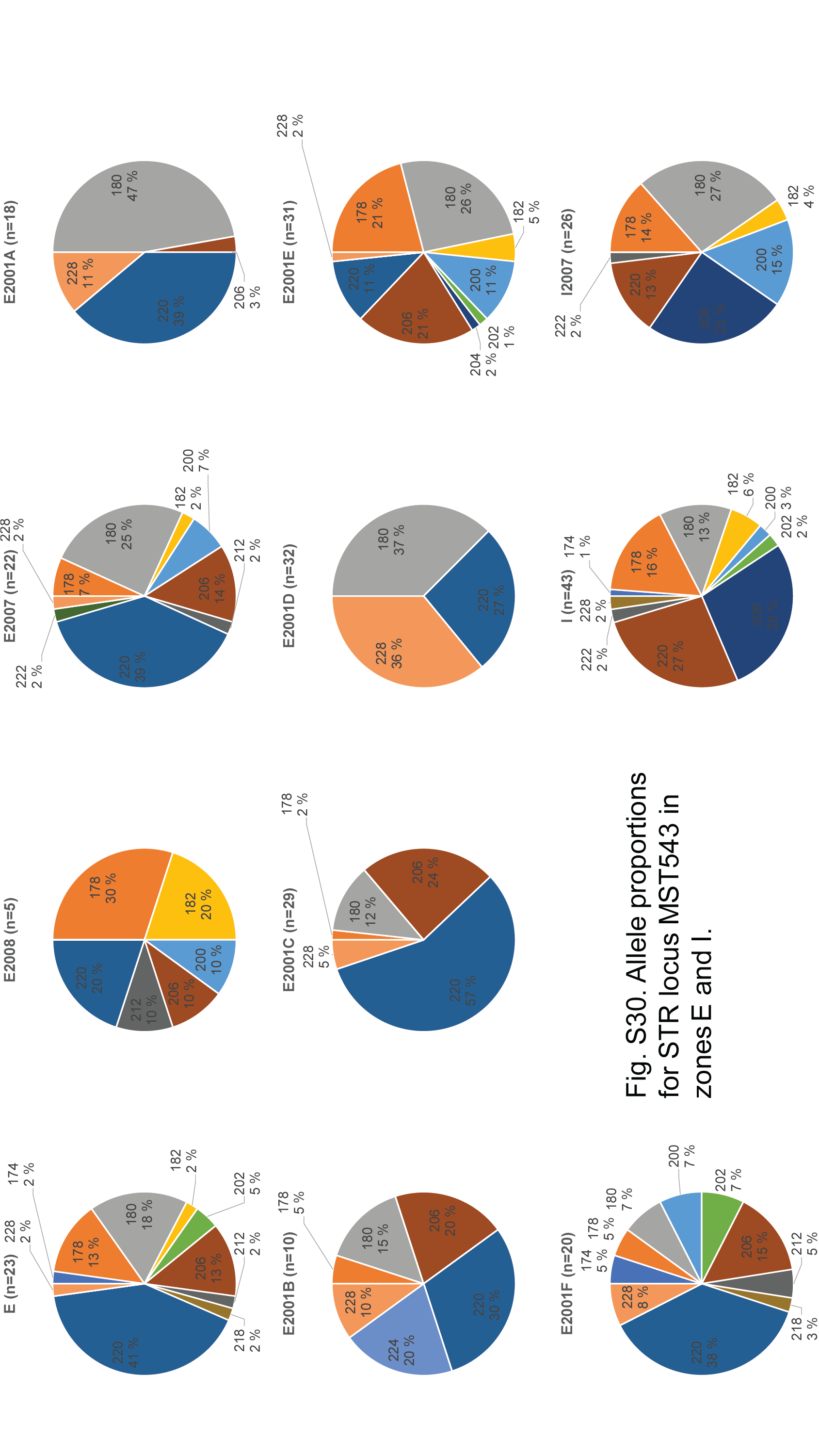
**

**Table S1**. Pairwise genetic differentiation (G_ST_) between river sections (below diagonal) and respective P values (above diagonal). Significant P values after a modified False Discovery Rate correction for multiple tests are indicated with a *. Bold highlighted P-values are significant from 0 (P < 0.01).

|  | **B** | **C** | **E** | **E2008** | **E2007** | **E2001**  **A** | **E2001**  **B** | **E2001**  **C** | **E2001**  **D** | **E2001**  **E** | **E2001**  **F** | **F** | **G** | **H** | **I** | **I2007** |
| --- | --- | --- | --- | --- | --- | --- | --- | --- | --- | --- | --- | --- | --- | --- | --- | --- |
| **B** | - | **0.000*** | **0.000*** | **0.000*** | **0.000*** | **0.000*** | **0.000*** | **0.000*** | **0.000*** | **0.000*** | **0.000*** | **0.000*** | **0.000*** | **0.000*** | **0.000*** | **0.000*** |
| **C** | 0.011 | - | 0.037* | **0.000*** | **0.000*** | **0.000*** | **0.000*** | **0.000*** | **0.000*** | **0.000*** | **0.000*** | **0.001*** | **0.000*** | **0.000*** | **0.000*** | **0.000*** |
| **E** | 0.027 | 0.004 | - | **0.001*** | **0.000*** | **0.000*** | **0.000*** | **0.000*** | **0.000*** | **0.000*** | 0.017* | 0.566 | **0.003*** | **0.000*** | **0.000*** | **0.000*** |
| **E2008** | 0.037 | 0.028 | 0.015 | - | 0.322 | **0.000*** | **0.000*** | **0.000*** | **0.000*** | **0.006*** | 0.051 | 0.024* | **0.005*** | 0.430 | 0.053 | 0.428 |
| **E2007** | 0.036 | 0.027 | 0.015 | 0.002 | - | **0.000*** | **0.000*** | **0.000*** | **0.000*** | **0.000*** | **0.003*** | **0.001*** | **0.000*** | **0.004*** | **0.000*** | 0.022* |
| **E2001A** | 0.096 | 0.089 | 0.072 | 0.056 | 0.047 | - | **0.000*** | **0.000*** | **0.000*** | **0.000*** | **0.000*** | **0.000*** | **0.000*** | **0.000*** | **0.000*** | **0.000*** |
| **E2001B** | 0.095 | 0.080 | 0.067 | 0.054 | 0.054 | 0.138 | - | **0.000*** | **0.000*** | **0.000*** | **0.000*** | **0.000*** | **0.000*** | **0.000*** | **0.000*** | **0.000*** |
| **E2001C** | 0.071 | 0.076 | 0.074 | 0.052 | 0.056 | 0.110 | 0.123 | - | **0.000*** | **0.000*** | **0.000*** | **0.000*** | **0.000*** | **0.000*** | **0.000*** | **0.000*** |
| **E2001D** | 0.078 | 0.060 | 0.047 | 0.065 | 0.054 | 0.134 | 0.113 | 0.093 | - | **0.000*** | **0.000*** | **0.000*** | **0.000*** | **0.000*** | **0.000*** | **0.000*** |
| **E2001E** | 0.042 | 0.030 | 0.017 | 0.010 | 0.014 | 0.060 | 0.065 | 0.073 | 0.063 | - | **0.000*** | **0.000*** | **0.000*** | **0.000*** | **0.000*** | **0.000*** |
| **E2001F** | 0.024 | 0.013 | 0.006 | 0.007 | 0.007 | 0.059 | 0.061 | 0.052 | 0.048 | 0.013 | - | 0.032* | **0.001*** | **0.008** | 0.011* | **0.000*** |
| **F** | 0.031 | 0.010 | -0.001 | 0.010 | 0.010 | 0.079 | 0.050 | 0.063 | 0.043 | 0.019 | 0.005 | - | 0.592 | 0.027* | 0.123 | **0.000*** |
| **G** | 0.031 | 0.014 | 0.006 | 0.009 | 0.015 | 0.081 | 0.054 | 0.059 | 0.046 | 0.017 | 0.006 | -0.001 | - | **0.007** | 0.032* | **0.000*** |
| **H** | 0.035 | 0.022 | 0.009 | 0.000 | 0.005 | 0.065 | 0.047 | 0.055 | 0.051 | 0.013 | 0.004 | 0.004 | 0.003 | - | 0.291 | **0.000*** |
| **I** | 0.032 | 0.018 | 0.011 | 0.005 | 0.011 | 0.077 | 0.047 | 0.057 | 0.053 | 0.013 | 0.005 | 0.002 | 0.003 | 0.001 | - | **0.000*** |
| **I2007** | 0.047 | 0.035 | 0.027 | 0.001 | 0.005 | 0.068 | 0.052 | 0.054 | 0.062 | 0.017 | 0.015 | 0.015 | 0.013 | 0.009 | 0.011 | - |

**Table S2.** Pairwise genetic differentiation (Jost’s D_EST_) between river sections (below diagonal) and respective P values (above diagonal). Significant P values after a modified False Discovery Rate correction for multiple tests are indicated with a *. Bold highlighted P-values are significant from 0 (P < 0.01).

|  | **B** | **C** | **E** | **E2008** | **E2007** | **E2001**  **A** | **E2001**  **B** | **E2001**  **C** | **E2001**  **D** | **E2001**  **E** | **E2001**  **F** | **F** | **G** | **H** | **I** | **I2007** |
| --- | --- | --- | --- | --- | --- | --- | --- | --- | --- | --- | --- | --- | --- | --- | --- | --- |
| **B** | - | **0.000*** | **0.000*** | **0.000*** | **0.000*** | **0.000*** | **0.000*** | **0.000*** | **0.000*** | **0.000*** | **0.000*** | **0.000*** | **0.000*** | **0.000*** | **0.000*** | **0.000*** |
| **C** | 0.035 | - | 0.036* | **0.000*** | **0.000*** | **0.000*** | **0.000*** | **0.000*** | **0.000*** | **0.000*** | **0.000*** | **0.001*** | **0.000*** | **0.000*** | **0.000*** | **0.000*** |
| **E** | 0.090 | 0.015 | - | **0.001*** | **0.000*** | **0.000*** | **0.000*** | **0.000*** | **0.000*** | **0.000*** | **0.017*** | 0.566 | **0.003*** | **0.000*** | **0.000*** | **0.000*** |
| **E2008** | 0.126 | 0.098 | 0.055 | - | 0.321 | **0.000*** | **0.000*** | **0.000*** | **0.000*** | **0.006** | 0.051 | 0.023* | **0.004*** | 0.429 | 0.052 | 0.427 |
| **E2007** | 0.122 | 0.095 | 0.056 | 0.007 | - | **0.000*** | **0.000*** | **0.000*** | **0.000*** | **0.000*** | **0.003*** | **0.001*** | **0.000*** | **0.004*** | **0.000*** | 0.022* |
| **E2001A** | 0.264 | 0.253 | 0.210 | 0.164 | 0.135 | - | **0.000*** | **0.000*** | **0.000*** | **0.000*** | **0.000*** | **0.000*** | **0.000*** | **0.000*** | **0.000*** | **0.000*** |
| **E2001B** | 0.272 | 0.236 | 0.204 | 0.165 | 0.165 | 0.348 | - | **0.000*** | **0.000*** | **0.000*** | **0.000*** | **0.000*** | **0.000*** | **0.000*** | **0.000*** | **0.000*** |
| **E2001C** | 0.188 | 0.213 | 0.214 | 0.150 | 0.164 | 0.259 | 0.304 | - | **0.000*** | **0.000*** | **0.000*** | **0.000*** | **0.000*** | **0.000*** | **0.000*** | **0.000*** |
| **E2001D** | 0.220 | 0.171 | 0.140 | 0.200 | 0.166 | 0.337 | 0.291 | 0.223 | - | **0.000*** | **0.000*** | **0.000*** | **0.000*** | **0.000*** | **0.000*** | **0.000*** |
| **E2001E** | 0.143 | 0.104 | 0.062 | 0.036 | 0.051 | 0.173 | 0.197 | 0.213 | 0.189 | - | **0.000*** | **0.000*** | **0.000*** | **0.000*** | **0.000*** | **0.000’** |
| **E2001F** | 0.085 | 0.048 | 0.022 | 0.025 | 0.029 | 0.177 | 0.194 | 0.155 | 0.149 | 0.048 | - | 0.032* | **0.001*** | **0.007*** | 0.011* | **0.000*** |
| **F** | 0.105 | 0.034 | -0.003 | 0.037 | 0.038 | 0.238 | 0.153 | 0.187 | 0.129 | 0.069 | 0.020 | - | 0.594 | 0.027* | 0.124 | **0.000*** |
| **G** | 0.103 | 0.049 | 0.023 | 0.034 | 0.054 | 0.237 | 0.162 | 0.171 | 0.137 | 0.059 | 0.024 | -0.002 | - | **0.007*** | 0.031* | **0.000*** |
| **H** | 0.121 | 0.079 | 0.032 | 0.001 | 0.018 | 0.194 | 0.144 | 0.162 | 0.158 | 0.050 | 0.017 | 0.014 | 0.012 | - | 0.291 | **0.000*** |
| **I** | 0.109 | 0.064 | 0.040 | 0.019 | 0.042 | 0.233 | 0.143 | 0.169 | 0.163 | 0.048 | 0.018 | 0.008 | 0.009 | 0.002 | - | **0.000*** |
| **I2007** | 0.159 | 0.124 | 0.100 | 0.004 | 0.018 | 0.199 | 0.158 | 0.157 | 0.189 | 0.061 | 0.059 | 0.055 | 0.049 | 0.033 | 0.040 | - |

**Additional references**

1. Cairney, M., Taggart, J. B. & Høyheim, B. Characterization of microsatellite and minisatellite loci in Atlantic salmon (*Salmo salar* L.) and cross‐species amplification in other salmonids. *Mol. Ecol*. **9(12),** 2175-2178 (2000).
2. Estoup, A., Presa, P., Krieg, F., Vaiman, D. & Guyomard, R. (CT)n and (GT)n microsatellites: a new class of genetic markers for *Salmo trutta* L. (brown trout). *Heredity* **71(5),** 488-496 (1993).
3. King, T. L., Eackles, M. S. & Letcher, B. L. Microsatellite DNA markers for the study of Atlantic salmon (*Salmo salar*) kinship, population structure, and mixed‐fishery analyses. *Mol. Ecol. Resour.* **5(1),** 130 – 132 (2005).
4. Olsen, J. B., Wilson, S. L., Kretschmer, E. J., Jones, K. C. & Seeb, J. E. Characterization of 14 tetranucleotide microsatellite loci derived from sockeye salmon. *Mol. Ecol.* **9(12),** 2185 – 2187 (2000).
5. Paterson, S., Piertney, S. B., Knox, D., Gilbey, J. & Verspoor, E. Characterization and PCR multiplexing of novel highly variable tetranucleotide Atlantic salmon (*Salmo salar* L.) microsatellites. *Mol. Ecol. Resour*. **4(2),** 160-162 (2004).
6. Poteaux, C., Bonhomme, F. & Berrebi, P. Microsatellite polymorphism and genetic impact of restocking in Mediterranean brown trout (*Salmo trutta* L.). *Heredity* **82,** 645–653 (1999).
7. Presa, P. & Guyomard, R. Conservation of microsatellites in three species of salmonids. *J. Fish Biol.* **49(6),** 1326-1329 (1996).
8. Rexroad, III C. E., Coleman, R. L., Hershberger, W. K. & Killefer, J. Eighteen polymorphic microsatellite markers for rainbow trout (*Oncorhynchus mykiss*). *Anim. Genet.* **33(1),** 76 – 78 (2002).
9. Klütsch, C. F. C., *et al.* Genetic changes caused by restocking and hydroelectric dams in demographically bottlenecked brown trout in a transnational subarctic riverine system. *Ecol. Evol.* **9(10)**, 6068-6081 (2019).
10. Puechmaille, S. J. The program structure does not reliably recover the correct population structure when sampling is uneven: subsampling and new estimators alleviate the problem. *Mol. Ecol. Resour.* **16(3)**, 608 – 627 (2016).
